# The ingenol-based protein kinase C agonist GSK445A is a potent inducer of HIV and SIV RNA transcription

**DOI:** 10.1371/journal.ppat.1010245

**Published:** 2022-01-18

**Authors:** Afam A. Okoye, Rémi Fromentin, Hiroshi Takata, Jessica H. Brehm, Yoshinori Fukazawa, Bryan Randall, Marion Pardons, Vincent Tai, Jun Tang, Jeremy Smedley, Michael Axthelm, Jeffrey D. Lifson, Louis J. Picker, David Favre, Lydie Trautmann, Nicolas Chomont

**Affiliations:** 1 Vaccine and Gene Therapy Institute, Oregon Health & Science University, Beaverton, Oregon, United States of America; 2 Oregon National Primate Research Center, Oregon Health & Science University, Beaverton, Oregon, United States of America; 3 Centre de Recherche du CHUM, Montréal, Québec, Canada; 4 Department of Microbiology, Infectiology and Immunology, Université de Montréal, Montreal, Québec, Canada; 5 ViiV Healthcare, Research Triangle Park, North Carolina, United States of America; 6 AIDS and Cancer Virus Program, Leidos Biomedical Research, Inc., Frederick National Laboratory, Frederick, Maryland, United States of America; 7 UNC HIV Cure Center, University of North Carolina at Chapel Hill, Chapel Hill, North Carolina, United States of America; 8 HIV Discovery Performance Unit, GlaxoSmithKline, Research Triangle Park, North Carolina, United States of America; Emory University, UNITED STATES

## Abstract

Activation of the NF-κB signaling pathway by Protein Kinase C (PKC) agonists is a potent mechanism for human immunodeficiency virus (HIV) latency disruption *in vitro*. However, significant toxicity risks and the lack of evidence supporting their activity *in vivo* have limited further evaluation of PKC agonists as HIV latency-reversing agents (LRA) in cure strategies. Here we evaluated whether GSK445A, a stabilized ingenol-B derivative, can induce HIV/simian immunodeficiency virus (SIV) transcription and virus production *in vitro* and demonstrate pharmacological activity in nonhuman primates (NHP). CD4^+^ T cells from people living with HIV and from SIV^+^ rhesus macaques (RM) on antiretroviral therapy (ART) exposed *in vitro* to 25 nM of GSK445A produced cell-associated viral transcripts as well as viral particles at levels similar to those induced by PMA/Ionomycin, indicating that GSK445A can potently reverse HIV/SIV latency. Importantly, these concentrations of GSK445A did not impair the proliferation or survival of HIV-specific CD8^+^ T cells, but instead, increased their numbers and enhanced IFN-γ production in response to HIV peptides. *In vivo*, GSK445A tolerability was established in SIV-naïve RM at 15 μg/kg although tolerability was reduced in SIV-infected RM on ART. Increases in plasma viremia following GSK445A administration were suggestive of increased SIV transcription *in vivo*. Collectively, these results indicate that GSK445A is a potent HIV/SIV LRA *in vitro* and has a tolerable safety profile amenable for further evaluation *in vivo* in NHP models of HIV cure/remission.

## Author summary

Antiretroviral therapy (ART) is not a definitive cure for HIV infection, in part, because the virus is able to integrate its genetic material in the host cell and remain in a dormant but fully replication-competent form during ART. These latently-infected cells can persist for long periods of time and remain hidden from the host’s immune system. If ART is stopped, the virus can reactivate from this pool of infected cells and resume HIV replication and disease progression. As such, finding and eliminating cells with latent HIV infection is priority for HIV cure research. One approach is to use compounds referred to as latency-reversing agents, that can induce HIV reactivation during ART. The goal of this approach is to facilitate elimination of infected cells by the virus itself once it reactivates or by the host’s immune system, once virus induction renders the cells detectable by the immune system, while also preventing the virus from infecting new cells due to the continued presence of ART. In this study we report on the activity of a novel latency-reversing agent called GSK445A, a potent activator of the enzyme protein kinase C (PKC). We show that GSK445A can induce HIV and simian immunodeficiency virus (SIV) latency reversal *in vitro* and has a tolerable saftey profile in nonhuman primates that should permit further testing of this PKC-agonist in strategies to cure HIV.

## Introduction

While ART effectively suppresses HIV replication, limiting disease progression and preventing transmission, it does not constitute definitive treatment for the infection, as virus persists despite ART, and recrudescent progressive disease ensues when treatment is stopped. Hence ART is not curative and in addition to side effects, ART does not optimally reconstitute the immune system of people with HIV (PWH) [1,2]. As such, novel immunotherapeutic strategies capable of inducing immune-mediated control of HIV replication in the absence of ART are needed. Inducing HIV expression during ongoing ART, combined with approaches for enhanced immune clearance of cells expressing induced viral antigens has been proposed as a strategy to eliminate persistently infected cells that could ultimately lead to viral eradication [3–5]. Several molecules with such potential for viral induction have been identified using *in vitro* models of HIV latency. They include Protein Kinase C (PKC) activators [6], histone deacetylase inhibitors [4,7–9], Toll-like receptor agonists [10–13] and common gamma-chain cytokines [14–16]. These molecules display highly variable activities between the models tested [17–19], modestly induce viral transcription *in vivo* [20,21], and have not resulted in a measurable decrease in the size of the HIV reservoir [4,22–24]. Among all the classes of latency reversing agents (LRAs) tested, PKC agonists have shown consistent latency-reversal activity in CD4^+^ T cells isolated from virally suppressed individuals [25].

Activation of the PKC pathway leads to induction of HIV transcription from latent proviral genomes through cooperative activation of NF-κB [6,26] and AP-1 [27,28]. Induction of viral transcription is associated with NF-κB nuclear translocation but also with increased expression of proteins involved in the positive transcription elongation factor b (P-TEFb) complex [29], a master regulator of HIV transcription [30]. In the past few years, several classes of PKC agonists have been developed: (i) phorbol esters; (ii) bryostatins and bryologs; and (iii) ingenol B and analogs. Those molecules have been evaluated for their ability to reverse latency in cell lines and in primary CD4^+^ T cells isolated from virally suppressed individuals [6,29,31–35]. Almost a decade ago, the non-tumor promoting phorbol ester prostratin was shown to activate latent HIV expression in PBMCs from ART-treated people [6]. Interestingly, prostratin can achieve viral reactivation without inducing cell proliferation *in vivo* [31]. Derivatives from the ingenol family demonstrate greater and more consistent latency reversal activity when compared to other LRAs in *ex vivo* models using CD4^+^ T cells from ART-suppressed individuals [29,35,36].

Concerns for a narrow therapeutic window with PKC agonists, have limited clinical trials. The PKC agonist bryostatin-1 has been administered to PWH [37]. However, although a single low dose administration of bryostatin-1 was well tolerated, it did not demonstrate any measurable impact on PKC activity nor on HIV transcription [37]. PKC agonists with a larger therapeutic window that can be administered safely at doses effective to reactivate the latent, replication-competent viral reservoir *in vivo* will be required to assess the utility of this intervention approach.

Studies of clonal expansions of T cells carrying replication-competent virus suggest that *in vivo*, cells harbouring HIV proviruses may expand without viral gene expression, and so may evade immune-mediated clearance [38,39]. Thus, an effective strategy to target the latent viral reservoir will likely depend on induction of viral expression from latent proviruses as well as immune clearance of cells with induced virus expression. However, it is important that candidate LRAs not impair immune responses. While it is well established that HIV-specific CD8^+^ T cells can play a crucial role in mediating antiviral immunity by killing productively infected CD4^+^ T cells, inhibition of CD8^+^ T cell responses might contribute to the observed lack of HIV reservoir reduction by the LRAs tested in clinical trials despite their ability to induce HIV gene expression [40,41]. Developing LRAs that can effectively reactivate latent proviruses while preserving CD8^+^ T cell responses will likely be required for HIV cure strategies aimed at targeting the replication competent viral reservoir using this approach. In vitro, histone deacetylase inhibitors inhibited proliferation of HIV-specific CD8^+^ T cells while PKC agonists showed an enhancement of this proliferation [40,41] suggesing that PKC agonists may have promise in this setting. Ingenol-B and other PKC agonists have been shown to lack the inhibitory effect of other LRAs on HIV-specific CD8^+^ T cell responses *in vitro* and may actually enhance HIV antigen processing and presentation to CD8^+^ T cells [41,42–44].

Here we assessed the latency reversal activity of a newly developed ingenol derivative, GSK445A [45]. Ex vivo models using PBMCs from ART suppressed PWH and SIV-infected rhesus macaques (RM) were used to evaluate the impact of GSK445A on P-TEFb activation and NF-kB phosphorylation, HIV/SIV transcription, HIV/SIV production and the functional activity of CD8^+^ T cells. In addition, GSK445A was administered to both SIV-naïve and SIV-infected RM on ART to assess tolerability and activity.

## Results

### GSK445A induces activation of cellular factors required for HIV/SIV reactivation from latency

**Fig 1:**
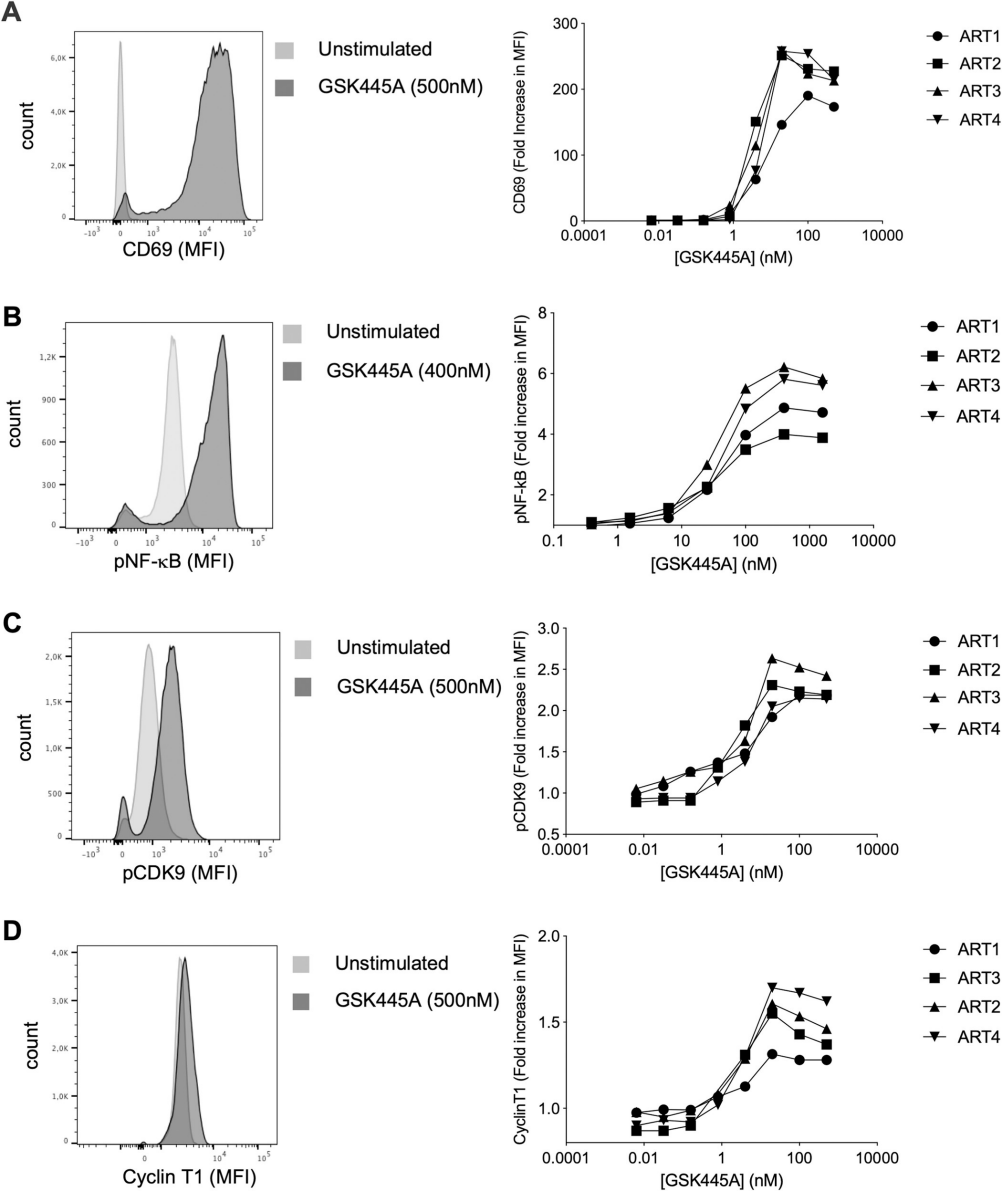
GSK445A induces cellular factors associated with HIV latency reversal. Expressions of cell surface CD69 (A), intracellular p-NFκB (pS529) (B), intracellular pCDK9 (pS175) and (C) intracellular cyclin T1 (D) following stimulation with increasing doses of GSK445A were measured by flow cytometry in CD4^+^ T cells from 4 HIV-infected and virally suppressed individuals. Representative histograms (left panels) and dose response curves (right panels) are shown.

We first assessed the ability of GSK445A to induce cellular factors required for HIV reactivation from latency ex vivo in CD4^+^ T cells from four virally suppressed individuals on ART. As expected, GSK445A induced CD69 expression on CD4^+^ T cells in a concentration dependent manner (**Fig 1A**), and this was accompanied by the phosphorylation of NF-kB, a transcription factor known to be activated upon activation of the PKC pathway (**Fig 1B**). Since positive transcription elongation factor (P-TEFb) has previously been shown to tightly regulate HIV transcription [30], we measured the ability of GSK445A to activate P-TEFb by measuring the expression of phosphorylated CDK9 as well as cyclin T1. Both these surrogates of P-TEFb activation were increased in a dose dependent manner following stimulation with GSK445A (**Fig 1C and 1D**). Importantly, similar results (increased CD69 expression, NF-kB phosphorylation and cyclin T1 expression) were obtained when CD4^+^ T cells from ART-suppressed SIV-infected RM were used (**S1A–S1C Fig**). Overall, the EC50s of GSK445A ranged from 5 to 50 nM depending on the readouts and were remarkably similar in human and RM CD4^+^ T cells (**S1D Fig**). Importantly, GSK445A resulted in minimal cell toxicity in both systems even when high concentrations of the compound were used (**S1E Fig**).

### GSK445A induces HIV/SIV latency reversal in purified CD4^+^ T cells from ART-suppressed PWH and RM

**Fig 2:**
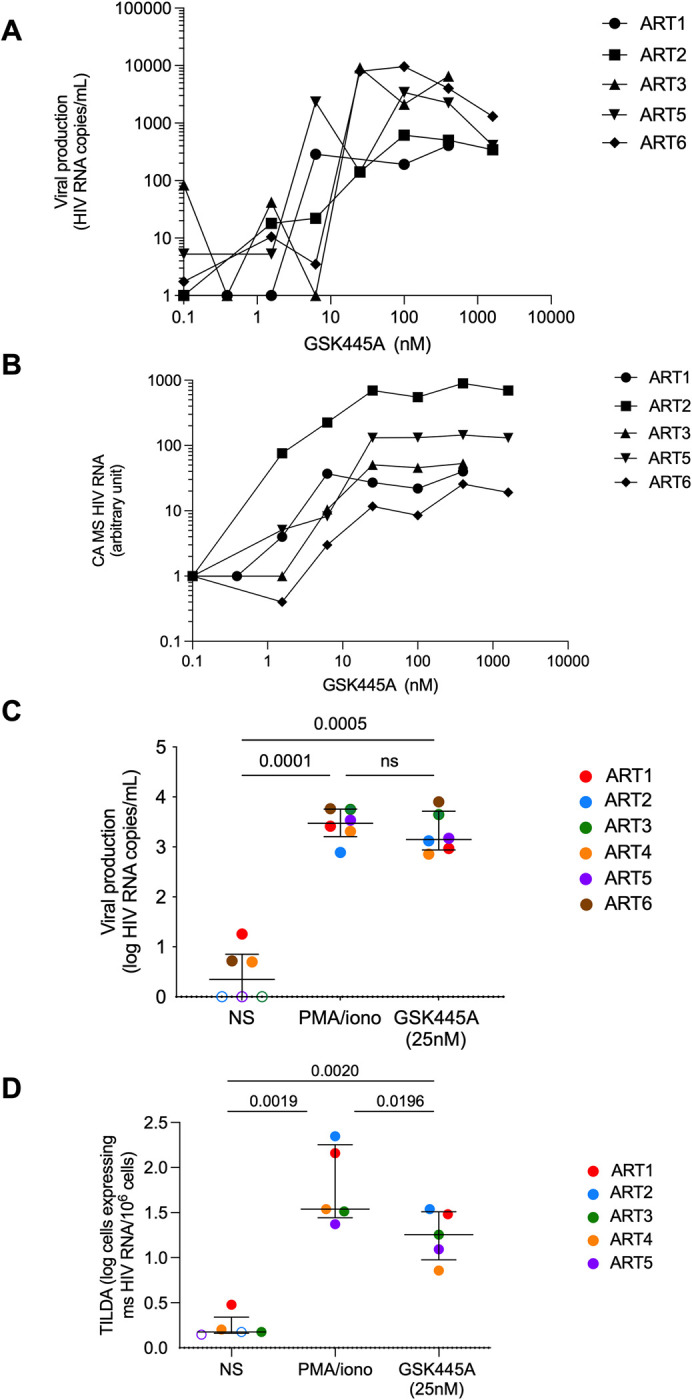
GSK445A induces HIV expression in CD4^+^ T cells from HIV-infected and virally suppressed individuals. **(**A) Free HIV particles were measured in the culture supernatants of CD4^+^ T cells obtained from 5 HIV-infected and virally suppressed individuals exposed to increasing doses of GSK445A. (B) Cell-associated multiply spliced HIV RNA (Tat/Rev) were measured in CD4^+^ T cells obtained from 5 HIV-infected and virally suppressed individuals exposed to increasing doses of GSK445A. (C) Viral production in culture supernatants of CD4^+^ T cells obtained from 6 HIV-infected and virally suppressed individuals and stimulated without (NS) or with PMA/ionomycin or GSK445A (25 nM). (D) Frequency of CD4^+^ T cells obtained from 5 HIV-infected and virally suppressed individuals producing Tat/Rev HIV transcripts when stimulated without (NS) or with PMA/ionomycin or GSK445A (25 nM). Frequencies of Tat/Rev^+^ cells were measured by TILDA. P values in C and D were obtained using one-way ANOVA with Tukey’s multiple-comparisons test.

Having demonstrated that GSK445A potently induces the expression of cellular factors that are critical to achieve HIV/SIV reactivation from latency, we directly assessed the capacity of this compound to induce viral production in latently infected CD4^+^ T cells from virally suppressed PWH. Initially, we performed a dose response experiment by exposing purified CD4^+^ T cells from 5 virally suppressed individuals to increasing doses of GSK445A. Although the amounts of viral particles released in the culture supernatant did not follow linear trends with increasing doses of the compound, GSK445A at concentrations above 10 nM consistently induced the production of HIV virions in the 5 samples tested (**Fig 2A**). However, measurements of cell-associated multiply spliced HIV RNA (Tat/Rev) showed a remarkable association between GSK445A concentrations and HIV transcriptional activity (**Fig 2B**). Of note, amounts of Tat/Rev transcripts reached maximal values at a concentration of 25 nM of GSK445A. We used this concentration in all subsequent experiments.

We then compared the amounts of viral particles produced by CD4^+^ T cells isolated from 6 virally suppressed individuals in response to GSK445A versus PMA/ionomycin. 25 nM of GSK445A induced levels of viral production similar to 162 nM PMA and 1 μg/mL ionomycin, demonstrating the potent latency reversal ability of the compound (**Fig 2C**). To measure the frequency of cells producing multiply spliced HIV RNA upon stimulation by GSK445A, we used the Tat/Rev Limiting Dilution Assay (TILDA) [46]. Although GSK445A reversed HIV latency in a smaller fraction of infected cells than PMA/ionomycin, it induced a significant increase in the frequency of cells producing multiply spliced HIV RNA when compared to the control condition (**Fig 2D**). Similarly, exposure of CD4^+^ T cells isolated from ART-suppressed RM to GSK445A resulted in the production of cell-associated SIV transcripts and viral particles (**S2A and S2B Fig**), indicating that the compound was also active on latent SIV. Altogether, these results identified GSK445A as a potent HIV/SIV LRA that is highly active in the nanomolar range.

### GSK445A synergistically increases the proliferation and function of TCR triggered HIV-specific CD8^+^ T cells

**Fig 3:**
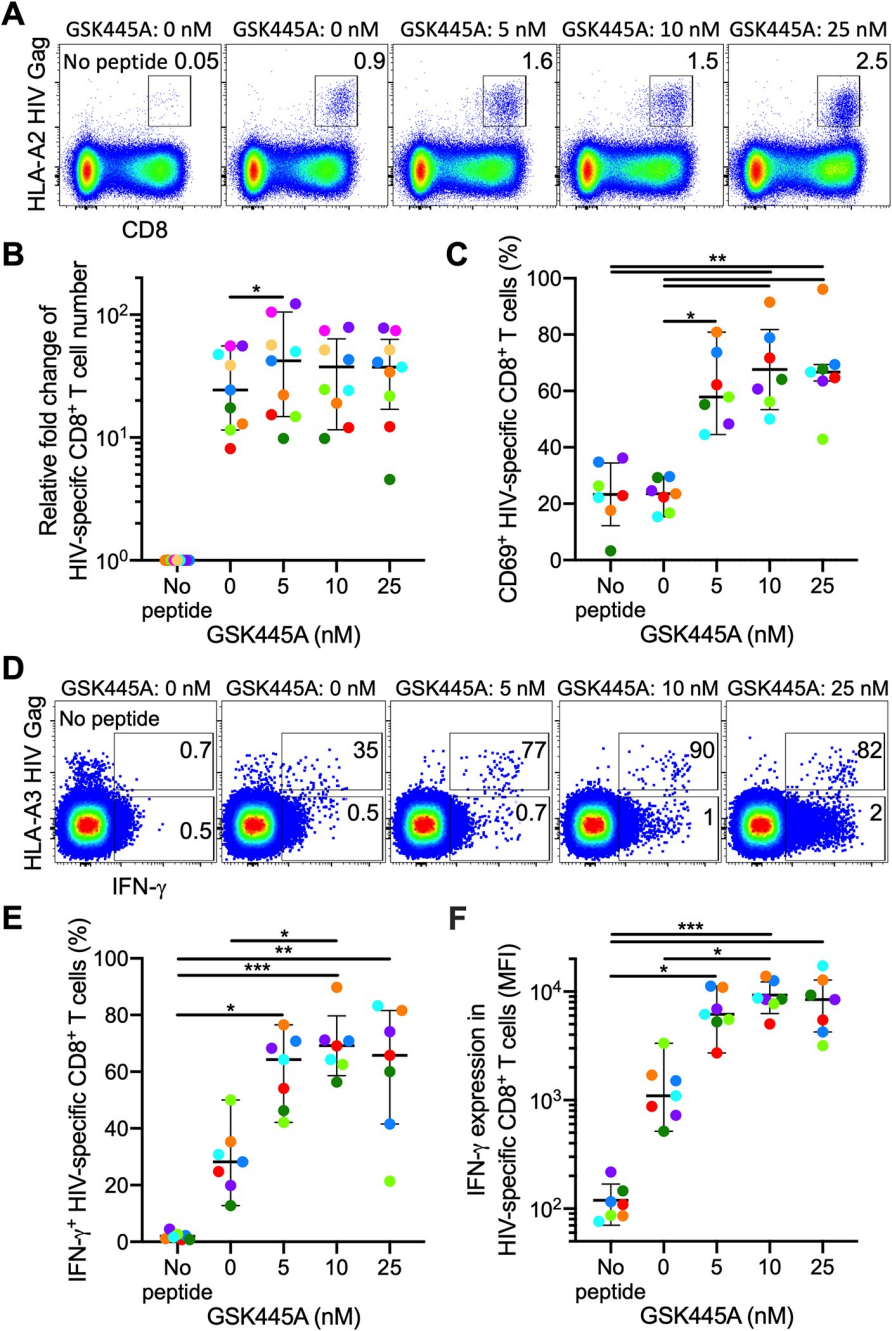
GSK445A synergistically enhances IFN-γ production in HIV-specific CD8^+^ T cells upon antigen stimulation. (A) Representative HLA-A0201 HIV Gag tetramer staining after 6 days of A2-Gag-FK10 peptide stimulation with prior 30 minutes GSK445A pulse. Percentage of tetramer^+^ CD8^+^ T cells in total CD8^+^ T cells is shown. (B) Fold change of HIV-specific CD8^+^ T cell numbers relative to the cells cultured without peptide and prior GSK445A pulse (No peptide) from 9 PWH are shown. (C) CD69 expression on HIV-specific CD8^+^ T cells from 7 PWH after 6 hours of the peptide stimulation with prior GSK445A pulse. (D) Representative expression of IFN-γ in HLA-A0301 tetramer^+^ HIV-specific CD8^+^ T cells or total CD8^+^ T cells after 6 hours of peptide stimulation. Percentage of IFN-γ^+^ cells within HIV-specific CD8^+^ T cells or total CD8^+^ T cells are shown. (E) Percentage of IFN-γ^+^ cells and (F) expression levels of IFN-γ (MFI) within HIV-specific CD8^+^ T cells from 7 PWH. Differences among conditions were analyzed with Friedman test followed by Dunn’s Multiple Comparisons test. *P< 0.05; **P< 0.01; ***P< 0.001.

For HIV cure strategies, LRAs need to reverse HIV latency without inhibiting HIV-specific CD8^+^ T cell responses to allow these cells to eliminate cells with induced viral expression. Since several classes of LRAs have been shown to be inhibitory to HIV-specific cytotoxic T cell responses [40], we evaluated the impact of GSK445A on the functional activity of HIV-specific CD8^+^ T cells. To assess this, we stimulated PBMCs from 9 PWH with HLA-A2-restricted FK10, HLA-A3-restricted RK10, HLA-B7-restricted TL10, or HLA-B8-restricted FL8 epitopes for 6 days after pulsing with increasing concentrations of GSK445A for 30 minutes. The stabilized ingenol-B derivative did not significantly inhibit the expansion of HIV-specific CD8^+^ T cells, and even significantly increased the number of cells at the 5 nM concentration (**Fig 3A and 3B**). These data indicate that GSK445A is not toxic to HIV-specific CD8^+^ T cells at concentrations that induce virus reactivation *in vitro*. To test the effect of GSK445A on antigen dependent IFN-γ induction by HIV-specific CD8^+^ T cells, we stimulated the PBMCs from 7 PWH for 6 hours with the same HIV peptides used for the proliferation assay. Treatment with GSK445A increased CD69 expression on both HIV-specific CD8^+^ T cells and total CD8^+^ T cells (**Figs 3C** and **S3A**). By measuring the number of tetramer^+^ HIV-specific CD8^+^ T cells producing IFN-γ after 6 hours stimulation, we observed that GSK445A increased the frequency of tetramer+ cells producing IFN-γ from 28% in the peptide alone condition to 69% and 66% in the conditions with peptide and GSK445A at 10 nM and 25 nM, respectively (**Fig 3D and 3E**). In contrast, IFN-γ production from total CD8^+^ T cells remained very low, less than 15% at the highest concentration of GSK445A (25 nM; **S3B Fig**). In line with the increase in the frequency of cells producing IFN-γ, we also observed a significant increase in the amount of IFN-γ produced per cell reflected in increased mean fluorescence intensity values (MFI) for intracellular cytokine staining (1396 MFI in the peptide alone condition to 9280 and 8674 MFI in the conditions with peptide and GSK445A at 10 nM and 25 nM, respectively) (**Fig 3F**). In contrast, IFN-γ MFI in total CD8^+^ T cells remained very low, less than 845 MFI at the highest concentration of GSK445A (**S3C Fig**). Altogether, these data suggest that GSK445A enhances IFN-γ production in antigen stimulated HIV-specific CD8^+^ T cells at concentrations that reactivate latent HIV in CD4^+^ T cells.

### GSK445A rapidly alters CD4^+^ T cell dynamics in SIV-naïve RM

**Fig 4:**
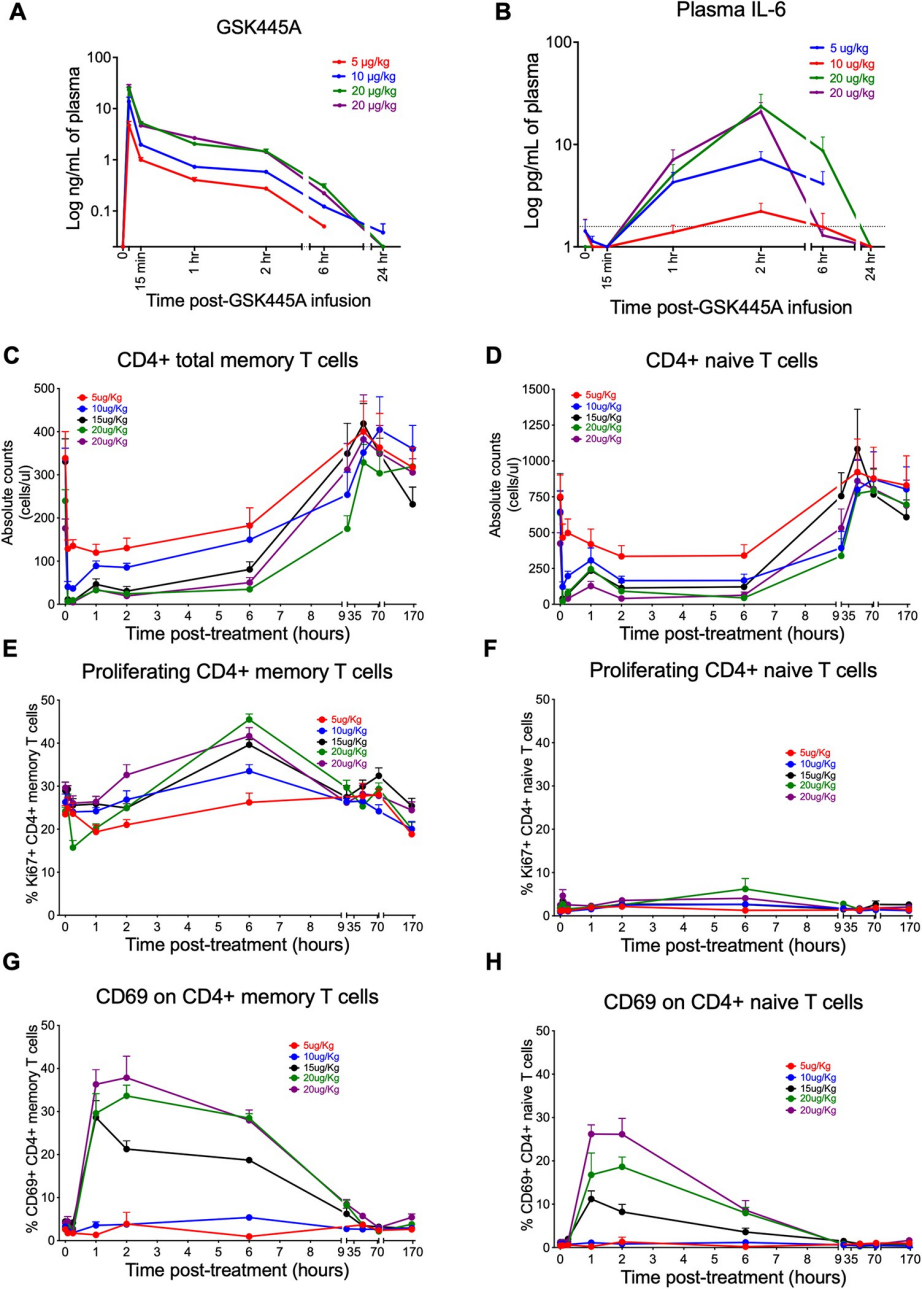
Effects of GSK445A on CD4^+^ T cell dynamics in SIV-naive RM. (A) Mean (+SEM) GSK445A drug levels in plasma of RM (n = 5) following 4 sequential intravenous infusions of GSK445A at 5 μg/kg, 10 μg/kg, 20 μg/kg and 20 μg/kg at 14-day intervals. (B) IL-6 levels in plasma of RM (n = 5) following sequential infusions of GSK445A. (C) Mean (+SEM) absolute counts of CD4^+^ memory T cells and (D) CD4^+^ naive T cells in blood of RM (n = 5) following sequential infusions of GSK445A at 5 μg/kg, 10 μg/kg, 20 μg/kg, 20 μg/kg and 15 μg/kg at 14-day intervals. (E) Mean (+SEM) frequencies of Ki67^+^ CD4^+^ memory T cells and (F) Ki67^+^ CD4^+^ naive T cells in blood of RM (n = 5) following sequential infusions of GSK445A at 5 μg/kg, 10 μg/kg, 20 μg/kg, 20 μg/kg and 15 μg/kg at 14-day intervals. (G) Mean (+SEM) frequencies of CD69^+^ CD4^+^ memory T cells and (F) CD69^+^ CD4^+^ naive T cells in blood of RM (n = 5) following sequential infusions of GSK445A at 5 μg/kg, 10 μg/kg, 20 μg/kg, 20 μg/kg and 15 μg/kg at 14-day intervals.

Having demonstrated the potency of GSK445A to induce HIV and SIV transcription *in vitro* with enhancing effects on the functional activity of CD8^+^ T cells, we next sought to characterize its activity *in vivo* in NHPs. In addressing this, we first performed a dose escalation study in healthy SIV naïve RM to identify a tolerable dose of GSK445A that induces CD4^+^ T cell activation, as measured by CD69 expression, with minimal toxicity. A total of 5 RM (**S1 Table**) received sequential intravenous infusions of GSK445A at 5 μg/kg, 10 μg/kg, 20 μg/kg and 20 μg/kg at 14-day intervals, with blood collected for pharmacokinetic (PK) and pharmacodynamic (PD) assessments. As shown in **Fig 4A**, GSK445A levels in plasma peaked 5 minutes post-infusion to a mean (+SEM) of 9.6 nM at the 5 μg/kg dose, 28 nM at the 10 μg/kg dose and 47.9nM or 53.3nM at the 20 μg/kg doses, respectively. Plasma GSK445A decay was biphasic, with an initial rapid decline by 15 minutes, followed by a slower 2^nd^ phase decay over the following 6 hours. GSK445A infusion was associated with a rapid increase in plasma IL-6 that peaked 2 hours post-infusion (**Fig 4B**), demonstrating IL-6 as a PD marker for GSK445A activity *in vivo*. Of note, 2 RM experienced clinical signs of erythema and mild respiratory distress after receiving the 20 μg/kg doses of GSK445A, but these were transient and quickly resolved. However, to determine whether a lower dose could mitigate these post-infusion reactions, RM received a 5^th^ infusion of GSK445A at 15 μg/kg and no clinical signs were observed.

Immediately following each infusion of GSK445A, there was a rapid, dose dependent decrease of naive and memory CD4^+^ T cells in blood (**Fig 4C and 4D**). The absolute numbers of naïve and memory CD8^+^ T cells, NK cells, B cells, monocytes and neutrophils in blood also declined rapidly immediately following treatment (**S4A–S4F Fig**). However, the lymphopenia was brief as most immune cell populations returned to baseline levels by 24 hours post-treatment, indicating exposure to GSK445A may induce a rapid but transient redistribution of cells from blood to tissues. This redistribution was not associated with high levels of homeostatic proliferation as the frequencies of proliferating (Ki67^+^) CD4^+^ memory and naive T cells increased briefly by 6 hours post-infusion but returned to baseline levels after 24 hours (**Figs 4E, 4F and S**5). In addition, no dramatic changes in the frequencies of proliferating CD8^+^ T cells or CD20^+^ B cells were observed post-infusion despite a similar loss in cells (**S6 Fig**).

We also explored the effect of GSK445A on CD69 expression on CD4^+^ T cells. As cell counts recovered, almost 30% (mean + SEM) of the reduced CD4^+^ memory T cell populaton present in blood (~14% of baseline) expressed CD69 by 1 hour following 15 μg/kg of GSK445A (**Figs 4G and S**7). Similarly, between 34–38% (mean + SEM) of the decreased population of CD4^+^ memory T cells present in blood (~14% - 19% of baseline) expressed CD69 by 2 hours following both doses of 20 μg/kg of GSK445A (**S7 and S8 Figs**). This phenomenon was not restricted to CD4^+^ memory T cells as we observed similar dynamics of increased CD69 expression on CD4^+^ naïve T cells, CD8^+^ T cells and CD20^+^ B cells as they reconstituted in blood following GSK445A infusion, particularly at doses above 15 μg/kg (**Figs 4H**, **S7** and **S9**). Note however that CD69 expression generally returned to pre-treatment levels after 24 hours, as cell counts normalized in blood. Interestingly, GSK445A had a dramatic effect on neutrophil counts, which increased rapidly and peaked between 2–6 hours post-infusion (**S4F Fig**), suggesting neutrophilia may be an additional PD marker of GSK445A activity *in vivo*.

Finally, we characterized the effects of GSK445A when infused over a shorter dosing interval. In assessing this we used the 15 μg/kg dose, which was the highest dose administered without clinical signs post-infusion. After a 4-week washout period, RM received 4 infusions of GSK445A at 15 μg/kg, twice a week on days 0, 3, 7 and 10. As previously observed, GSK445A induced a rapid but transient decrease in T cell counts in blood immediately following each infusion. As peripheral blood cell counts began to normalize, increased frequencies of CD69 expression were seen on the reduced CD4^+^ and CD8^+^ memory T cells in blood, an effect that peaked by 2 hours post-infusion (**S10A Fig**). Expression of the proliferation marker Ki67 on the reduced population of memory T cells in blood increased initially from a mean (+SEM) of 19.9% to 42.6% for CD4^+^ T cells and a mean (+SEM) of 14.9% to 30% for CD8^+^ T cells, but only after the first dose (**S10B Fig**). Subsequently, the proportion of proliferating T cells remained steady despite additional doses. Importantly, no clinical symptoms were observed post-infusion, indicating that short-interval dosing of GSK445A at 15 μg/kg was well tolerated in SIV naïve RM.

### GSK445A also alters CD4^+^ T cell dynamics in SIV-infected RM on ART

**Fig 5:**
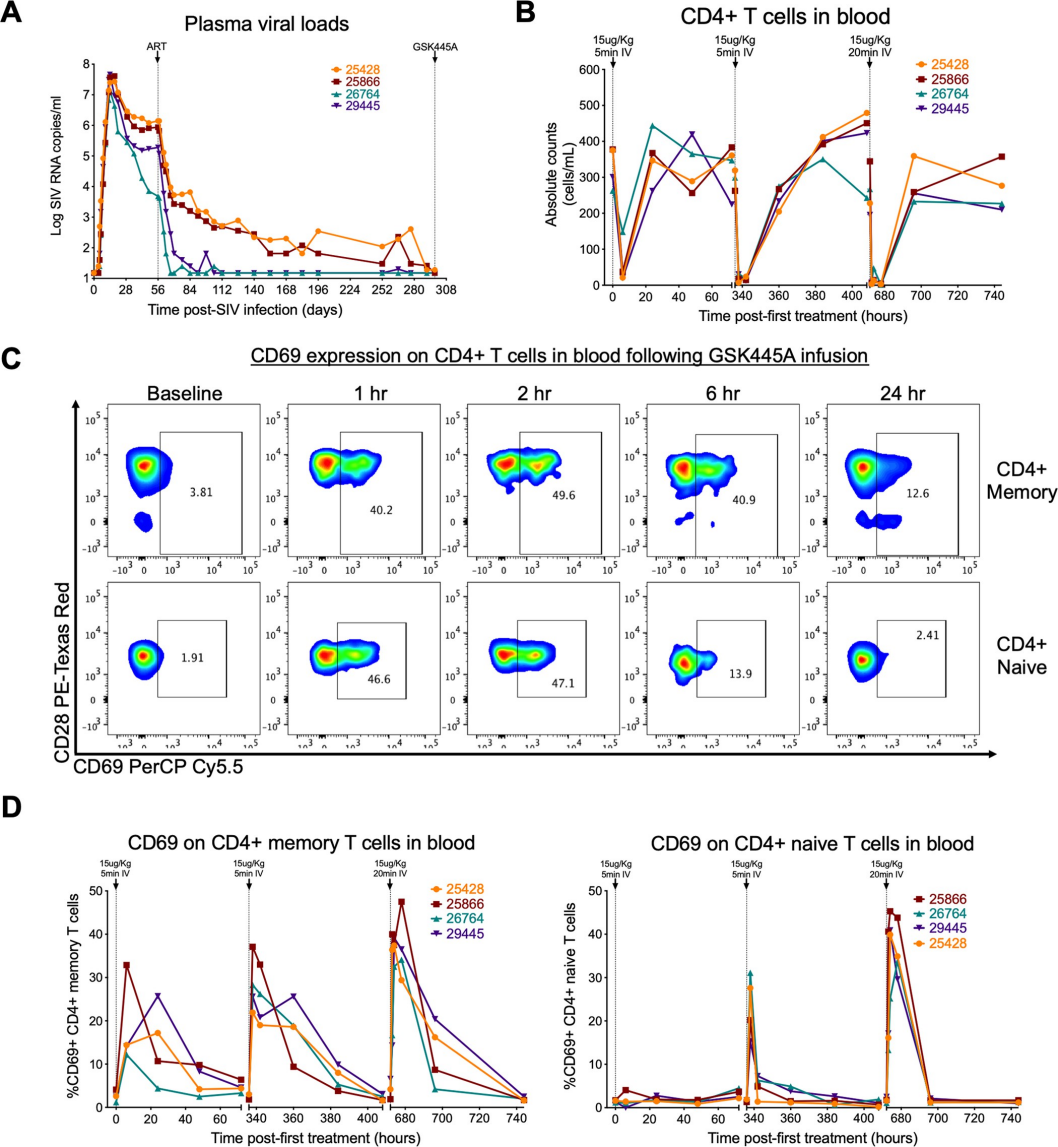
Effects of GSK445A on CD4^+^ T cell dynamics in SIV-infected RM on ART. (A) Plasma SIV RNA profiles for 4 RM following SIVmac239M infection, ART initiation on day 56 post-infection (pi) and GSK445A infusion starting day 298 pi. (B) Absolute counts of CD4^+^ T cells in blood following 3 infusions of GSK445A at 15 μg/kg. Note that the first 2 doses of GSK445A were administered over 5 minutes while the 3rd dose was administered over 20 minutes. (C) Representative flow cytometric analysis of RM RM25866 showing CD69 expression on CD4^+^ memory and naïve T cells in peripheral blood up to 24 hours following the third infusion of GSK445A at 15 μg/kg. (D) Frequencies of CD69^+^ CD4^+^ memory T cells (left panel) and CD69^+^ CD4^+^ naïve T cells (right panel) in blood following GSK445A infusion.

Next, we assessed the tolerability of GSK445A in SIV-infected RM on suppressive ART consisting of tenofovir disoproxil, emtricitabine and dolutegravir, using the non-toxic dose of 15 μg/kg that was tolerated by SIV uninfected RM. To address this we inoculated 4 RM with SIVmac239M followed by ART initiation 56 days post-infection (**S2 Table**). After 34 weeks of ART suppression (**Fig 5A**), RM received 3 infusions of GSK445A at 15 μg/kg each at 14-day intervals. Interestingly, 2 SIV^+^ RM on ART were noted with signs of erythema of the facial area and mild respiratory distress following 15 μg/kg of GSK445A, suggesting tolerability is reduced in ART-suppressed RM. In order to mitigate these clinical signs, we administered a 3^rd^ infusion of GSK445A at the same dose over 20 minutes in contrast to the 5-minute infusion used for the previous 2 doses. Although post-infusion reactions still occurred, all clinical signs completely resolved within 1 hour of treatment without any long-term sequelae. As previously described, GSK445A induced a rapid but transient decline in the absolute numbers of CD4^+^ T cells in blood immediately following each infusion (**Fig 5B**). Following the rapid decrease in cell populations in blood, we again observed increased frequencies of CD69 expression on the reduced populations of CD4^+^ memory and naïve T cells remaining in blood, with slightly higher fractions of CD69^+^ cells observed following the 3^rd^ infusion (**Figs 5C, 5D and S**11).

**Fig 6:**
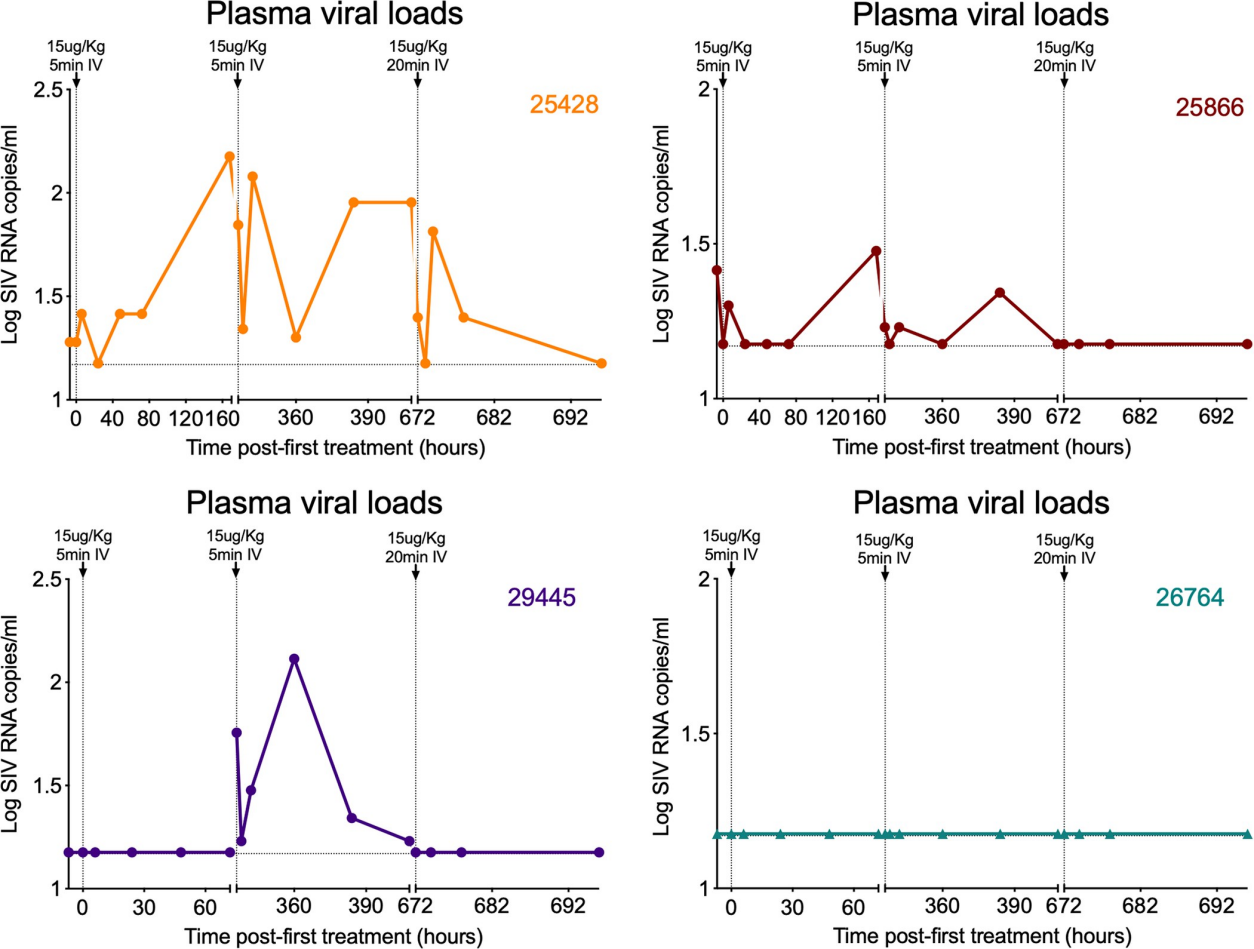
Effects of GSK445A on plasma viral loads. Plasma viral loads profiles of 4 RM following 3 infusions of GSK445A at 15 μg/kg. Limit of detection was 15 RNA copies/ml of plasma.

Despite the small number of RM in this study (n = 4), we performed preliminary analyses to determine whether GSK445A infusion has any effect on SIV dynamics in SIV-infected RM on ART. Of note, 3 of 4 RM showed measurable increases in SIV RNA in plasma above the threshold of 15 RNA copies/ml following GSK445A infusion, although both the frequency and the magnitude of transient viremia were variable (**Fig 6**). While these results are suggestive of increased SIV production in response to GSK445A infusion, the small sample size and variability in plasma viral loads at time of ART initiation are a limitation. Indeed RM26764, which had no increase in plasma viremia following GSK445A infusion, also had the lowest level of plasma viremia at time of ART initiation (**Fig 5A**), consistent with a smaller reservoir [47]. It is likely that RM with smaller replication-competent reservoirs may require more doses of GSK445A to observe a measurable increase in plasma viremia. We next quantified cell-associated viral loads in blood and observed transient but variable changes in levels of SIV RNA and SIV DNA in PBMC normalized to CD4^+^ T cells after GSK445A infusion in all 4 RM (**S12 Fig**). In some RM, there appeared to be modest increases in SIV RNA following GSK445A infusion that would indicate increased SIV transcription. Levels of SIV DNA also appeared to increase over time, which may be associated with the redistribution of cells from tissues into the periphery following GSK445A-induced lymphopenia. However, it is important to note that interpretation of these results are complicated by the dynamic treatment related changes in the peripheral blood cell populations sampled for these analyses over the time the samples were obtained. Hence, it remains to be determined whether the increase in plasma viral loads observed following GSK445A infusion was a direct result of the induction of SIV transcripton in latently-infected cells *in vivo*, or as an indirect effect of GSK445A-induced immune activation. To address this, it would be crucial to perform these studies in a larger cohort of RM with uniformly calibrated reservoirs and tightly defined post-infusion sampling of both peripheral blood and tissues, in order to fully characterize the *in vivo* LRA activity of GSK445A. However, these data do indicate that GSK445A at a dose of 15 μg/kg is relatively tolerable in SIV-infected RM on ART. In addition, they provide evidence to suggest that GSK445A may induce SIV expression in RM during ART, preliminary findings which support the further evaluation of GSK445A as an LRA for use *in vivo*.

## Discussion

Kick and kill strategies aim at forcing viral gene expression to reduce the size of the persistent viral reservoir through viral cytotoxicity and/or immune-mediated killing. Proof-of-concept clinical studies using HDAC inhibitors, disulfiram (DSF) [48] or toll-like receptor agonists [24] have shown transient increases in cell-associated HIV RNA, but had limited or no impact on the size of the viral reservoir. Novel, and possibly more potent LRAs, such as bromodomain inhibitors (iBET), IL-15, TLR7 and TLR9 agonists, immune checkpoint blockers (anti-PD-1) are currently being tested [49]. Among all classes of LRAs, PKC agonists have been described as the most potent stimulators of HIV expression *ex vivo* [19] and represent attractive candidates for kick and kill strategies if side effects and toxicity are controlled [6,29,31–35]. Currently, developing and progressing safe and effective single or combination strategies in the clinic remains challenging due to inherent general toxicity on other non-targeted cells, tissues and/or organs. In this study, we demonstrate that the stabilized ingenol-B derivative GSK445A is a potent HIV/SIV LRA in CD4^+^ T cells isolated from virally suppressed human and RM. Importantly, GSK445A did not impair CD8^+^ T cell responses *ex vivo* and induced SIV production *in vivo* in the majority of the animals evaluated.

Bryostatin-1 is the only PKC agonist that has been evaluated for its PK and cytotoxicity in humans, but the doses used were minimal and likely not have been sufficient to exert HIV proviral reactivation activity [37]. GSK445A has nanomolar range activity was tolerated by RM at doses able to induce SIV expression. Our *ex vivo* experiments revealed that GSK445A potently induces the activation of several cellular factors that have been identified as critical for efficient HIV/SIV transcription, including NF-κB and P-TEFb. Nonetheless, GSK445A may not counteract other factors involved in the transcriptional and translational repression of proviruses, including histone acetylation, limitation of export of HIV transcripts from the nucleus and degradation of viral transcripts by miRNA. Indeed, using TILDA, we observed that GSK445A induces the production of Tat/Rev transcripts in a relatively modest fraction of CD4^+^ T cells compared to PMA/ionomycin. In addition, different cellular reservoirs may show different sensitivities to a particular LRA, as suggested in two recent studies [50,51]. Therefore, combining GSK445A with other LRAs may be required to effectively reverse latency in all subsets of CD4^+^ T cells. Combinations between a PKC agonist and other LRAs have been tested *in vitro* and resulted in enhanced viral reactivation [36,52–55]. This could be tested *in vivo* in future studies. Combination would have the other advantage of lowering the dose of GSK445A administered without loosing efficacy of reactivation, potentially improving the tolerability of the drug.

In addition to its ability to safely reactivate latent proviruses, GSK445A enhanced the proliferation and effector function of HIV-specific CD8^+^ T cells in our *in vitro* assays. We noted an increase in the frequency of HIV-specific CD8^+^ T cells producing IFNγ as well as a notable higher production of IFNγ per cell following antigen stimulation in the presence of GSK445A, more than we’ve previously observed with immune checkpoint blockade [56]. Further studies would be needed to confirm whether GSK445A can also increase HIV-specific CD8^+^ T cell responses *in vivo*, as the timing of CD8^+^ T cell exposure to HIV antigens *in vivo* might be different from the peak exposure to GSK445A, which has a short window of bioactivity. However, even some exposure to the PKC agonist could prepare the CD8^+^ T cells to be better activated following recognition of reactivating HIV-infected CD4^+^ T cells. The ability of GSK445A to induce the upregulation of CD69 and redistribution of CD8^+^ T cells to tissues could also have a positive impact on the elimination of HIV infected cells by CD8^+^ T cells.

Our *in vivo* experiments identified 15 μg/kg as a dose of GSK445A that could be given to RM safely and repeatedly. GSK445A infusion induced a rapid decline in peripheral blood cell counts, possibly associated with a redistribution of cells from blood to tissues. A similar effect has been observed following the administration of gamma-chain cytokines such as IL-7, with lymphocyte migration driven by an IL-7-dependent increase in chemokine receptor expression on circulating T cells and the initiation of chemokine transcription in tissues [57]. The rapid loss in cells from blood suggests GSK445A exposure may transiently alter the homing potential of circulating lymphocytes. Cell loss coincided with high expression of CD69 on the decreased populations of CD4^+^ T cells, CD8^+^ T cells and CD20^+^ B cells remaining in blood. Subsequently, we found GSK445A activity had some effects on SIV dynamics in SIV-infected RM on ART. Indeed, despite moderate toxicity at the 15 μg/kg dose of GSK445A, 3 of 4 SIV^+^ RM on ART showed measurable increases in SIV viremia.

While our studies demonstrate the potency of GSK445A as an LRA *in vitro* and potential LRA activity *in vivo*, a few important issues remain regarding the development of GSK445A and other PKC agonists for use in the clinic; 1) Although we were able to observe increases in plasma viremia following 3 doses of GSK445A, its effectiveness to induce SIV transcription from latently-infected cells *in vivo*, particularly in lymphoid compartments such as lymph nodes, spleen and the intestinal mucosa, remains unclear. 2) GSK445A also impacted the activation status of B cells, which can be latently-infected with gammaherpes viruses such as Epstein-Barr virus (EBV) and Kaposi’s sarcoma-associated herpesvirus (KSHV), both of which have mechanisms that target NF-κB during oncogenesis [58,59]. Hence, GSK445A’s ability to modulate the NF-κB pathway could enhance the pathogenesis of these oncogenic viruses. This can be assessed in RMs by evaluating the impact of GSK445A on simian homologues of EBV and KHSV, rhesus lymphocryptovirus and rhesus rhadinovirus, respectively [60–63]. 3) Although 15 μg/kg of GSK445A was well tolerated in SIV naïve RM, tolerability was reduced in SIV-infected RM on ART. Although all clinical signs of treatment related adverse events resolved quickly post-GSK445A infusion, the tolerability of longer-term dosing (3–6 months) remains unclear and suggests further optimization is required in the context of ART-suppressed SIV infection. 4) To reduce toxicity, an option would be to use a lower dose of GSK445A in combination with other LRAs. As previously mentioned, PKC agonists have been shown to synergize with bromodomain inhibitors and histone deacetylase inhibitors to increase HIV transcription and virus production in CD4^+^ T cell from ART-suppressed patients [53, 64]. However, whether PK and PD considerations can allow these agents to synergize with GSK445A *in vivo* to reverse latency remains to be determined.

A number of agents have been assessed for their LRA activity in NHP models with most showing limited ability to increase virus production *in vivo* [65–69]. However, the second mitochondrial-derived activator of caspases (SMAC) mimetic AZD5582 was shown to have potent LRA activity via the activation of the non-canonical NF-κB pathway [70]. The *in vivo* activity of AZD5582 is quite different from GSK445A, with the former inducing SIV production with minimal CD4^+^ T cell activation, although increases in T cell proliferation were observed. In addition, the timing of virus reactivation following AZD5582 dosing was quite variable with some monkeys not showing increases in plasma viral loads until after 2 or 3 doses. Of note, data on the effect of AZD5582 on HIV/SIV-specific CD8^+^ T cell function are not yet available. In comparison, GSK445A appears to be more rapid in its ability to induce virus production *in vivo*, although at the expense of global lymphocyte activation, which increases the risk of toxicity. The long-term utility of both agents for “kick and kill” studies, particularly when used in combination with effector molecules such as broadly neutralizing antibodies or therapeutic vaccines will be important to assess. We believe these results provide strong rationale to support the further development of GSK445A and other PKC agonists for use in NHP models of HIV cure/remission and for potential clinical use in PWH if safety concerns can be addressed.

## Materials & methods

### Ethics statement

The human biological samples were sourced ethically and their research use was in accordance with the terms of the informed consents under an IRB/EC protocol approved by the Royal Victoria Hospital and the CHUM hospital (QC, Canada) review boards (FWA #00004139). All studies were conducted in accordance with the GSK Policy on the Care, Welfare and Treatment of Laboratory Animals and were reviewed by the Institutional Animal Care and Use Committee either at GSK or by the ethical review process at the institution where the work was performed; the Oregon National Primate Research Center’s Animal Care and Use Committee, under the standards of the US National Institutes of Health Guide for the Care and Use of Laboratory Animals.

### Study population

HIV-seropositive individuals (n = 6) on stable suppressive ART were enrolled in this study. The characteristics of the participants can be found in **S3 Table**. All participants signed inform consent approved by the Royal Victoria Hospital and the CHUM hospital (QC, Canada) review boards (FWA #00004139). Participants underwent leukapheresis to collect large numbers of PBMCs.

### Rhesus macaques

A total of 9 purpose-bred male RMs (*Macaca*. *mulatta*) of Indian genetic background were used for these experiments. These RMs were specific pathogen-free as defined by being free of cercopithecine herpesvirus 1, D-type simian retrovirus, simian T-lymphotropic virus type 1, rhesus rhadinovirus, and *Mycobacterium tuberculosis*. For the dose finding study, 5 male RM (**S1 Table**) received 5 μg/kg, 10 μg/kg, 20 μg/kg, 20 μg/kg and 15 μg/kg, of GSK445A administered intravenously (IV) by a 5-minute infusion at 14-day intervals. After a 4-week washout, these 5 RM received 4 additional doses of GSK445A, IV at 15 μg/kg, twice a week for 2 weeks. A second cohort of 4 male RM were IV inoculated with 100 or 200 infectious units (IU) of SIVmac239M and placed on ART starting 56 dpi (**S2 Table**). The SIVmac239M challenge stock used in this experiment was produced in transfected HEK-239T cells and the stock infectivity titer was determined using TZM-bl cells as previously described [71]. The daily ART regimen consisted of subcutaneous injection of 5.1 mg kg^−1^ d^−1^ tenofovir disoproxil, 40 mg kg^−1^ d^−1^ emtricitabine and 2.5 mg kg^−1^ d^−1^ dolutegravir in a solution containing 15% (v/v) kleptose at pH 4.2, as previously described [47]. At 298 dpi (242 days post-ART), RM received 3 doses of GSK445A at 15 μg/kg administered IV by 5-minute infusion for the first 2 doses and 20-minute infusion for the 3^rd^ dose.

### Isolation of human and macaque total CD4^+^ T cells and culture

Total CD4^+^ T cells were isolated from cryopreserved PBMCs using magnetic negative selection kit (StemCell Technology, Vancouver, Canada). Total CD4^+^ T cells were exposed to GSK445A for 30 minutes, washed and cultured for a total of 18 hours in the presence of antiretroviral drugs (200 nM raltegravir, 200 nM 3TC). 162 nM PMA and 1 μg/mL ionomycin stimulation was used as a positive control for reactivation.

### Quantification of plasma GSK445A and IL-6

Whole blood from ACD tubes was centrifuged to obtain plasma for PK and cytokine profiling. GSK445A concentration was determined from plasma by LC-MS/MS analysis (Frontage). Plasma concentrations of NHP IL-6 was determined according the manufacturer’s recommendations for the V-PLEX Proinflammatory Panel 1 NHP Kit (Meso Scale Discovery) using a 1:1 dilution of NHP plasma and Diluent 2 plus 1% Triton-X100. IL-6 was measured on a Meso Scale Discovery Sector S 600 plate reader and analysis carried out with MSD Discovery Workbench v4.0.12 and GraphPad PRISM 6.

### Quantification of Tat/Rev inducible multiply spliced HIV RNA

Total CD4^+^ T cells were used to measure the frequency of CD4^+^ T cells with inducible multiply spliced HIV RNA using the Tat/Rev induced limiting dilution assay (TILDA) [46].

### Quantification of cell-associated unspliced LTR-Gag and multiply-spliced Tat/Rev HIV/SIV RNA

Cell-associated RNAs were extracted using the RNeasy kit (Qiagen). Extracted RNA was reverse transcribed and subjected to 16 cycles of amplification using the primers listed in **S4 Table**. Preamplified products were diluted and subjected to a nested real-time PCR for 40 cycles on the Rotor-Gene Q by using the primers and probes listed in **S4 Table**. In all experiments, serial dilutions of ACH-2 cells or 3D8 cells were processed in parallel of experimental samples and were used to generate standard curves.

### Quantification of cell-free HIV/SIV RNA

Freshly collected cell culture supernatants were ultracentrifuged for 1 hour at 25,000 g to pellet HIV particles. By using this procedure, only packed viral RNAs were quantified, excluding HIV/SIV RNAs that are passively released in the medium as a consequence of cell death. Viral RNAs were extracted using the Qiamp viral RNA kit (Qiagen) and quantified using an ultrasensitive semi-nested real time RT-PCR, as described previously [72].

### Quantification of SIV plasma viral loads

Plasma SIV RNA levels were determined using a gag-targeted quantitative real time/digital RT-PCR format assay, essentially as previously described, with 6 replicate reactions analyzed per extracted sample for assay threshold of 15 SIV RNA copies/ml [73]. Samples that did not yield any positive results across the replicate reactions were reported as a value of “less than” the value that would apply for one positive reaction out of 6 [73].

### Quantification of cell-associated SIV RNA and SIV DNA

Quantitative assessment of SIV DNA and RNA in PBMC was performed using gag targeted nested quantitative hybrid real-time/digital RT-PCR and PCR assays, as previously described [73,74]. SIV RNA or DNA copy numbers were normalized based on quantitation of a single copy rhesus genomic DNA sequence from the CCR5 locus from the same specimen to allow normalization of SIV RNA or DNA copy numbers per 10^6^ diploid genome cell equivalents, as described [75]. Ten replicate reactions were performed with aliquots of extracted DNA or RNA from each sample, with two additional spiked internal control reactions performed with each sample to assess potential reaction inhibition. Samples that did not yield any positive results across the replicate reactions were reported as a value of “less than” the value that would apply for one positive reaction out of 10. Threshold sensitivities for individual specimens varied as a function of the number of cells and analyzed. To normalize cell-associated viral loads in PBMC to per 10^6^ CD4^+^ T cells, the % CD3^+^ CD4^+^ T cells in each sample analyzed was determined by flow cytometry and divided by SIV RNA and DNA copy numbers.

### Antibodies

For *ex vivo* experiments aimed at measuring cellular factors involved in HIV/SIV reactivation, the following reagents were used: Live/Dead Aqua Cell Stain (405 nm) was obtained from ThermoFisher Scientific (L34957). Anti-CD3 SP34.2 A700 (557917), anti-CD3 UCHT1 BUV496 (564809), anti-CD3 UCHT1 A700 (557943), anti-CD4 L200 BUV395 (564107), anti-CD4 RPA-T4 BV421 (562424), anti-CD8 RPA-T8 Pac Blue (558207), anti-CD8 RPA-T8 BUV395 (563795) and anti-pS529 NF-kB PE (558423) were obtained from BD Biosciences. Anti-CD69 FN50 FITC (310904) was purchased from BioLegend. Anti-CD4 S3.5 Qdot605 (Q10008) was obtained from Thermofisher. Anti-Cyclin T1 (sc-8127 TRITC) was obtained from Santa Cruz. Anti-pS175 CDK9 was kindly provided by Merck. Anti-KC57 PE (6604667) was obtained from Beckman Coulter and anti-p24 28B7 APC (MM-0289-APC) was obtained from MediMabs.

To measure the impact of GSK445A on HIV-specific CD8^+^ T cells, the following antibodies were used: Anti-CD3 (UCHT1: Alexa700; BD Biosciences), anti-CD4 (RPA-T4: Brilliant Violet 605; BioLegend), anti-CD8 (RPA-T8: Brilliant Violet 785; BioLegend), anti-CD69 (FN50: PerCP Cy5.5; BioLegend), anti-IFN-γ (4S.B3: Pacific Blue; BioLegend). HLA-A*0201 restricted HIV-1 Gag 433–442 (FK10, FLGKIWPSYK), HLA-A*0301 restricted Gag 20–28 (RK9, RLRPGGKKK), HLA-B*0702 restricted Nef 128–137 (TL10, TPGPGVRYPL), and HLA-B*0801 HIV-1 Nef 90–97 (FL8, FLKEKGGL) soluble biotinylated monomers were produced as previously described [76] at Vaccine and Gene Therapy Institute Florida (Port St. Lucie, FL) and NIH Tetramer Core Facility (Atlanta, GA) respectively. They were tetramerized with PE-conjugated Extravidin (Sigma-Aldrich). LIVE/DEAD Fixable Aqua Dead Cell Stain Kit (LIVE/DEAD) was from Thermo Fisher Scientific (Molecular Probes).

For *in vivo* experiments, combinations of fluorochrome-conjugated monoclonal antibodies used for staining included anti-CD3 SP34-2 BUV395 (564117), anti-CD4 L200 BVU786 (563914), anti-CD8α SK1 BUV737 (612754), anti-CCR5 3A9 APC (624046), anti-*K*i67 B56 FITC (Custom Bulk 624046), anti-CD14 M5E2 FITC (624046), anti-CD20 L27 APC-Cy7 (Custom Bulk 655118) and anti-streptavidin BV421 (563259) obtained from BD Biosciences. Anti-CD95 DX2 PE (Custom Bulk CUST00525), anti-CD69 CH/4 PerCP-Cy5.5 (MHCD6918) and obtained from Life Technologies. Anti-CD56 MEM-188 PerCP-Cy5.5 (MHCD5618CS3), anti-CD28 CD28.2 PE-Texas Red (PE-CF594) (302942), anti-CD16 3G8 BV650 (302042) and anti-HLA-DR L243 PE-CF594 (307654) obtained from BioLegend. Anti-NKG2A REA110 APC (130-095-212) obtained from Miltenyi and anti-CCR7 15053 Biotin (MAB197) obtained from R&D Systems.

### Immunophenotyping

For *in vivo* experiments to determine the phenotype of lymphocyte populations, whole blood was stained for flow-cytometric analysis as previously described [77–79]. Polychromatic (8–14 parameter) flow-cytometric analysis was performed on an LSR II BD instrument using Pacific blue, BUV395, BUV737, BV421, BV510, BV605, BV711, BV786, FITC, PE, PE-Texas red (PE-CF594), PE-Cy7, PerCP-Cy5.5, APC, APC-Cy7, and A700 as the available fluorescent parameters. Instrument setup and data acquisition procedures were performed as previously described [77–79]. List mode multiparameter data files were analyzed using the FlowJo software program (Tree Star). Criteria for delineating T_N_ and T_M_ subsets and for setting positive (+) versus negative (−) markers for CCR5 and Ki-67 expression have been previously described in detail [77–79]. In brief, T_N_ constitute a uniform cluster of cells with a CD28^moderate^, CCR7^+^, CCR5^−^, CD95^low^ phenotype, which is clearly distinguishable from the phenotypically diverse memory population that is CD95^high^ or displays one or more of the following non-naive phenotypic features: CD28^−^, CCR7^−^, CCR5^+^. For delineating NK cells in blood, small lymphocytes were gated to obtain CD3^-^ CD8α^+^ NKG2A^+^ cells that were CD20^-^ and CD14^-^ as previously described [80]. Similarly, B cells were delineated gating on small lymphocytes to obtain CD3^-^ CD20^+^ cells. For each subset to be quantified, the percentages of the subset within the overall small lymphocyte and/or small T cell (CD3^+^ small lymphocyte) populations were determined. For quantification of peripheral blood subsets, absolute small lymphocyte counts were obtained using an AcT5diff cell counter (Beckman Coulter) and from these values, absolute counts for the relevant subset were calculated based on the subset percentages within the light scatter–defined small lymphocyte population on the flow cytometer. Absolute counts of monocytes and neutrophils were obtained directly from the AcT5diff cell counter.

### NF-κB activation

Blood mononuclear cells were rested for 3 hours (RM cells) or 14 hours (human cells) and exposed to increasing doses of GSK445A for 20 minutes (37°C). LIVE/DEAD staining was performed at the same time than the stimulation. Cells were then fixed with Cytofix Fixation Buffer (BD Biosciences, 554655) (10 minutes, 37°C) and stained with anti-CD3 SP34.2 A700, CD4 L200 BUV395, CD8 RPA-T8 PB for RM cells, and with anti-CD3 UCHT1 A700, CD4 S3.5 Qdot605, CD8 RPA-T8 PB for human cells (45 minutes, RT). Cells were permeabilized for 20 minutes on ice with ice-cold Permeabilization Buffer III (BD Biosciences, 558050), and rehydrated in PBS/Human Serum 10% (Atlanta Biologicals, 540110) for 15 minutes on ice. Finally, PBMCs were stained (30 minutes, RT) with anti-pS529 NF-κB antibody. Data were collected on an LSR-II flow cytometer (BD Biosciences) and analysis performed using FlowJo software (Tree Star).

### P-TEFb activation and CD69 expression

Blood mononuclear cells were rested for 8 hours, incubated for 20 minutes on ice and exposed to increasing doses of GSK445A for 18 hours. Cells were then stained (30 minutes, 4°C) with the Aqua LIVE/DEAD staining kit, and anti-CD3 SP34.2 A700, anti-CD4 L200 BUV395, anti-CD8 RPA-T8 PB, anti-CD69 FN50 FITC for RM cells, and anti-CD3 UCHT1 BUV496, anti-CD4 S3.5 Qdot605, anti-CD8 RPA-T8 PB and anti-CD69 FN50 FITC for human cells. After a 20 minute-fixation with Methanol free Formaldehyde 4% (Polysciences, 04018), cells were permeabilized (30 minutes, 4°C) with the Perm Wash buffer (BD Biosciences, 554723), and stained (30 minutes, RT) with anti-pS175 CDk9 and anti-Cyclin T1 antibodies. Data were collected on an LSR-II flow cytometer (BD Biosciences) and analysis performed using FlowJo software (Tree Star).

### Impact of GSK445A on HIV-specific CD8^+^ T cells

Freshly thawed PBMCs from PWH were treated with GSK445A at 37°C for 30 minutes in RPMI-1640 containing 10% fetal bovine serum (R10). GSK445A treated cells were washed twice in R10, and cultured for 6 days in RPMI-1640 containing with 8% human serum (Access Biologicals), recombinant human IL-2 (20U/mL, Miltenyi Biotech), and HLA-A*0201 restricted HIV-1 Gag 433–442 (FK10, FLGKIWPSYK), HLA-A*0301 restricted Gag 20–28 (RK9, RLRPGGKKK), HLA-B*0702 restricted Nef 128–137 (TL10, TPGPGVRYPL), or HLA-B*0801 restricted HIV-1 Nef 90–97 (FL8, FLKEKGGL) peptide (1μg/ml, AnaSpec). Cultured PBMCs were stained with PE-labeled pMHC tetramer in PBS containing 2% fetal bovine serum (FACS buffer) at 37°C for 15 minutes. The cells were stained for the other cell surface markers at 4°C for 20 minutes followed by 2 times wash with FACS buffer, and then fixed with PBS containing 2% formaldehyde. CountBright beads (Thermo Fisher Scientific) were added to the samples before flow cytometry analysis, and the tetramer^+^ cell number was normalized based on recorded number of the CountBright beads acquired on the LSRII (BD Biosciences).

For intracellular IFN-γ staining, GSK445A-treated PBMCs were stimulated with the same HIV cognate peptides above (1 μg/ml) for 7 hours, with GolgiPlug (BD Biosciences) added for the last 6 hours of stimulation. Stimulated cells were stained with PE-labeled pMHC tetramer and the other cell surface markers before fixation with PBS containing 2% formaldehyde. Cells were permeabilized in Perm/Wash Buffer (BD Biosciences) at room temperature for 15 minutes after 20 minutes fixation. The cells were stained with anti-IFNγ Ab at room temperature for 30 minutes followed by 3 washes with FACS buffer, and then fixed with PBS containing 2% formaldehyde. Data were collected on an LSR-II flow cytometer (BD Biosciences) and analysis performed using FlowJo software (Tree Star).

### Statistics

Levels of viral induction (viral production and TILDA) were log-transformed and analyzed using one-way ANOVA with Tukey’s multiple-comparisons test. Differences between immunological parameters were analyzed with Friedman test followed by Dunn’s Multiple Comparisons test. All statistical analyses were performed with GraphPad Prism 8.0. p values < 0.05 were considered statistically significant.

## Supporting information

S1 Table Characteristics of SIV naïve animals. (TIF)

S2 Table Characteristics of SIV-infected animals. (TIF)

S3 Table Characteristics of the study participants. (TIF)

S4 Table Sequences of the primers and probes used in the CA US HIV RNA, CA MS HIV RNA and CF HIV RNA. (TIF)

S1 Fig GSK445A shows similar activities in cells from humans and RM. Expressions of cell surface CD69 (A), intracellular p-NFκB (B) and intracellular cyclin T1 (C) following stimulation with increasing doses of GSK445A were measured by flow cytometry in CD4^+^ T cells from 4 RM. Representative histograms (left panels) and dose response curves (right panels) are shown. D: EC50 of GSK5445A for induction of CD69, pNF-κB and cyclin T1. (E) Cellular toxicity of GSK445A was evaluated by exposing increasing doses of the compound to isolated CD4^+^ T cells from 3 virally suppressed individuals (left panel) and 4 RM (right panel).
(TIF)

S2 Fig GSK445A induces SIV expression in CD4^+^ T cells from virally suppressed RM. (A) Unspliced SIV RNA (gag) in CD4^+^ T cells obtained from 3 virally suppressed RMs and stimulated without (NS) or with GSK445A (25 nM). (B) Viral production in culture supernatants of CD4^+^ T cells obtained from 3 virally suppressed RMs and stimulated without (NS) or with GSK445A (25 nM).
(TIF)

S3 Fig Effect of GSK445A treatment on total human CD8^+^ T cells. (A) CD69 expression on tetramer^-^ total CD8^+^ T cells from 7 PWH after 6 hours of peptides stimulation with prior GSK445A pulse. Percentage of IFN-γ^+^ cells (B) and expression levels of IFN-γ (C) within total CD8^+^ T cells. Differences among conditions were analyzed by Friedman test. *P< 0.05; **P< 0.01; ***P< 0.001; ****P< 0.0001.
(TIF)

S4 Fig GSK445A induces rapid but transient depletion of lymphocytes in RM. (A-F) Mean (+SEM) absolute counts of CD8^+^ memory T cells, CD8^+^ naïve T cells, NK cells, CD20^+^ B cells, monocytes and neutrophils in blood of RM (n = 5) following sequential intravenous infusions of GSK445A at 5 μg/kg, 10 μg/kg, 20 μg/kg, 20 μg/kg and 15 μg/kg at 14-day intervals.
(TIF)

S5 Fig Effects of GSK445A on CD4^+^ memory and naïve T cell proliferation. Absolute counts and frequencies of Ki67^+^ CD4^+^ memory T cells (left panels) and Ki67^+^ CD4^+^ naïve T cells (right panels) in blood of RM following sequential intravenous infusions of GSK445A at 5 μg/kg, 10 μg/kg, 20 μg/kg, 20 μg/kg and 15 μg/kg at 14-day intervals.
(TIF)

S6 Fig Effects of GSK445A on CD8^+^ T cells and CD20^+^ B cells in blood of SIV naïve RM. Mean (+SEM) frequencies of Ki67^+^ CD8^+^ memory T cells, Ki67^+^ CD8^+^ naïve T cells and Ki67^+^ CD20^+^ B cells in blood of RM (n = 5) following sequential intravenous infusions of GSK445A at 5 μg/kg, 10 μg/kg, 20 μg/kg, 20 μg/kg and 15 μg/kg at 14-day intervals.
(TIF)

S7 Fig Effects of GSK445A on CD4^+^ memory and naïve T cell activation. Absolute counts and frequencies of CD69^+^ CD4^+^ memory T cells (left panels) and CD69^+^ CD4^+^ naïve T cells (right panels) in blood of RM following sequential intravenous infusions of GSK445A at 5 μg/kg, 10 μg/kg, 20 μg/kg, 20 μg/kg and 15 μg/kg at 14-day intervals.
(TIF)

S8 Fig Showing increased frequencies of CD69^+^ CD4^+^ T cells in blood following GSK445A infusion. Representative flow cytometric analysis of an SIV-naïve RM showing CD69 expression on CD4^+^ memory and naïve T cells in peripheral blood following infusion of GSK445A at 20 μg/kg.
(TIF)

S9 Fig Effects of GSK445A on CD8^+^ T cells and CD20^+^ B cell activation. Absolute counts and frequencies of CD69^+^ CD8^+^ memory T cells (left panels), CD69^+^ CD8^+^ naïve T cells (middle panels) and CD69^+^ CD20^+^ B cells (right panels) in blood of RM following sequential intravenous infusions of GSK445A at 5 μg/kg, 10 μg/kg, 20 μg/kg, 20 μg/kg and 15 μg/kg at 14-day intervals.
(TIF)

S10 Fig Effects of biweekly GSK445A dosing on CD4^+^ and CD8^+^ T cells in blood of SIV naïve RM. (A) Mean (+SEM) absolute counts and frequencies of CD69^+^ CD4^+^ memory T cells (left panel) and CD69^+^ CD8^+^ memory T cells (right panel) in blood of RM (n = 5) following 4 biweekly doses of GSK445A at 15 μg/kg. (B) Mean (+SEM) frequencies Ki67^+^ CD4^+^ memory T cells (left panel) and CD8^+^ memory T cells (right panel) in blood of RM (n = 5) following 4 biweekly doses of GSK445A at 15 μg/kg.
(TIF)

S11 Fig Effects of GSK445A on CD4^+^ memory and naïve T cells in blood of SIV^+^ RM on ART. Absolute counts and frequencies of CD69^+^ CD4^+^ memory T cells (left panels) and CD69^+^ CD4^+^ naïve T cells (right panels) in blood of 4 SIV-infected RM on ART following 3 doses of GSK445A infusion at 15 μg/kg.
(TIF)

S12 Fig Longitudinal assessment of SIV DNA and SIV RNA in PBMC of SIV^+^ RM on ART. Cell-associated SIV DNA (left panel) and SIV RNA (right panel) in PBMC normalized to CD4^+^ T cells in each sample analyzed, from 4 SIV-infected RM on ART following 3 doses of GSK445A at 15 μg/kg. Cell-associated SIV RNA and SIV DNA were assessed by qRT-PCR and qPCR, respectively.
(TIF)
