# Supplementary figures and images for: The ingenol-based protein kinase C agonist GSK445A is a potent inducer of HIV and SIV RNA transcription
Source: PLoS Pathog. 2022 Jan 18;18(1):e1010245. doi: 10.1371/journal.ppat.1010245 (PMC8797195; doi:10.1371/journal.ppat.1010245)

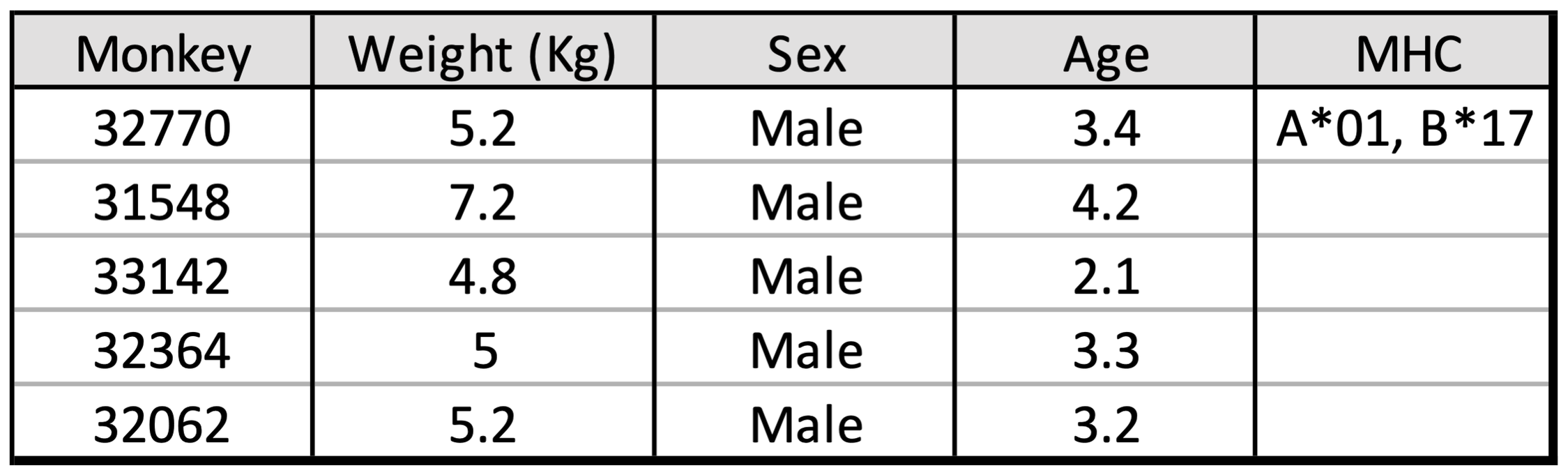

Supplement: S1 Table — (TIF) [file ppat.1010245.s001.tif]

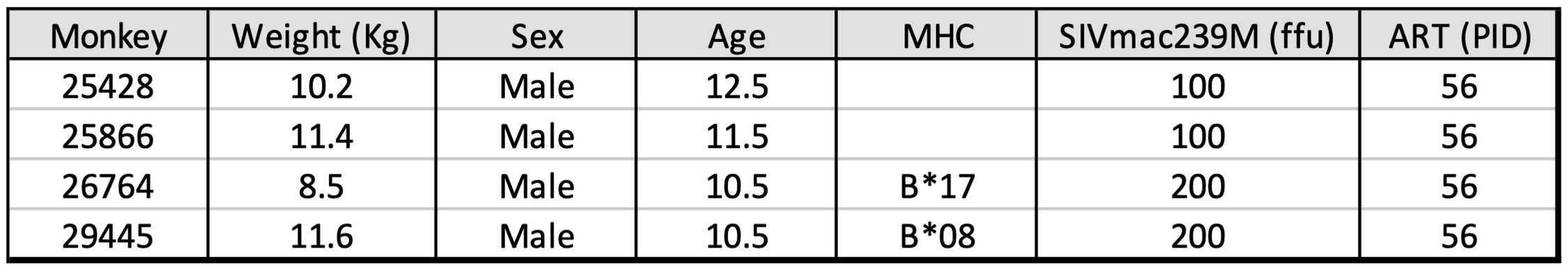

Supplement: S2 Table — (TIF) [file ppat.1010245.s002.tif]

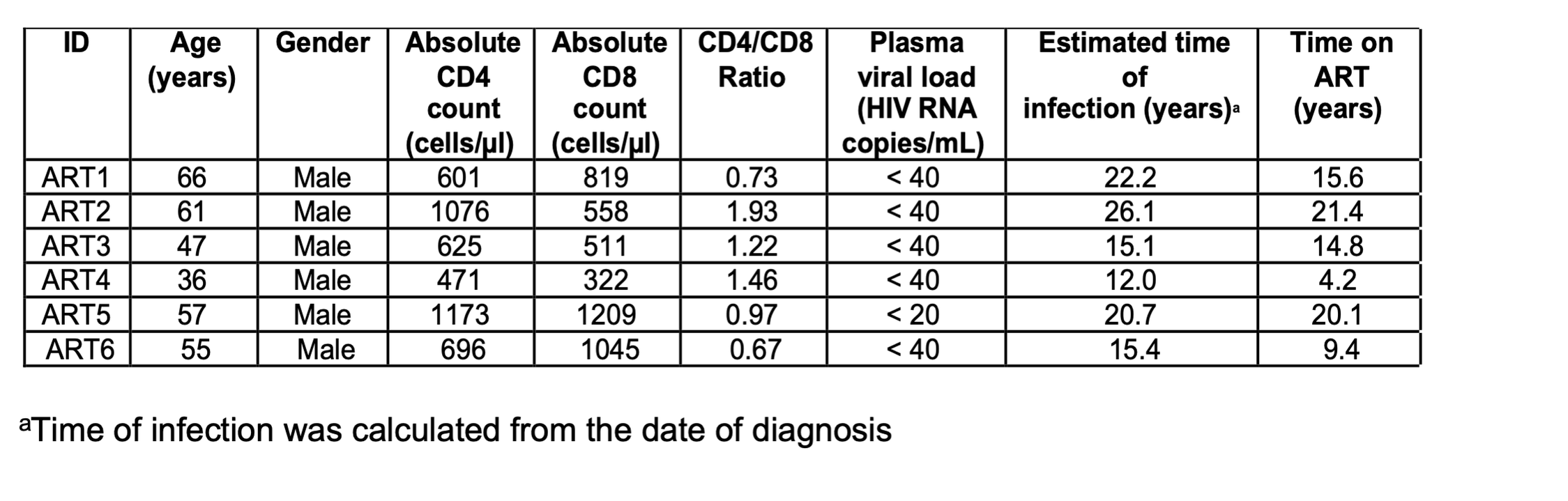

Supplement: S3 Table — (TIF) [file ppat.1010245.s003.tif]

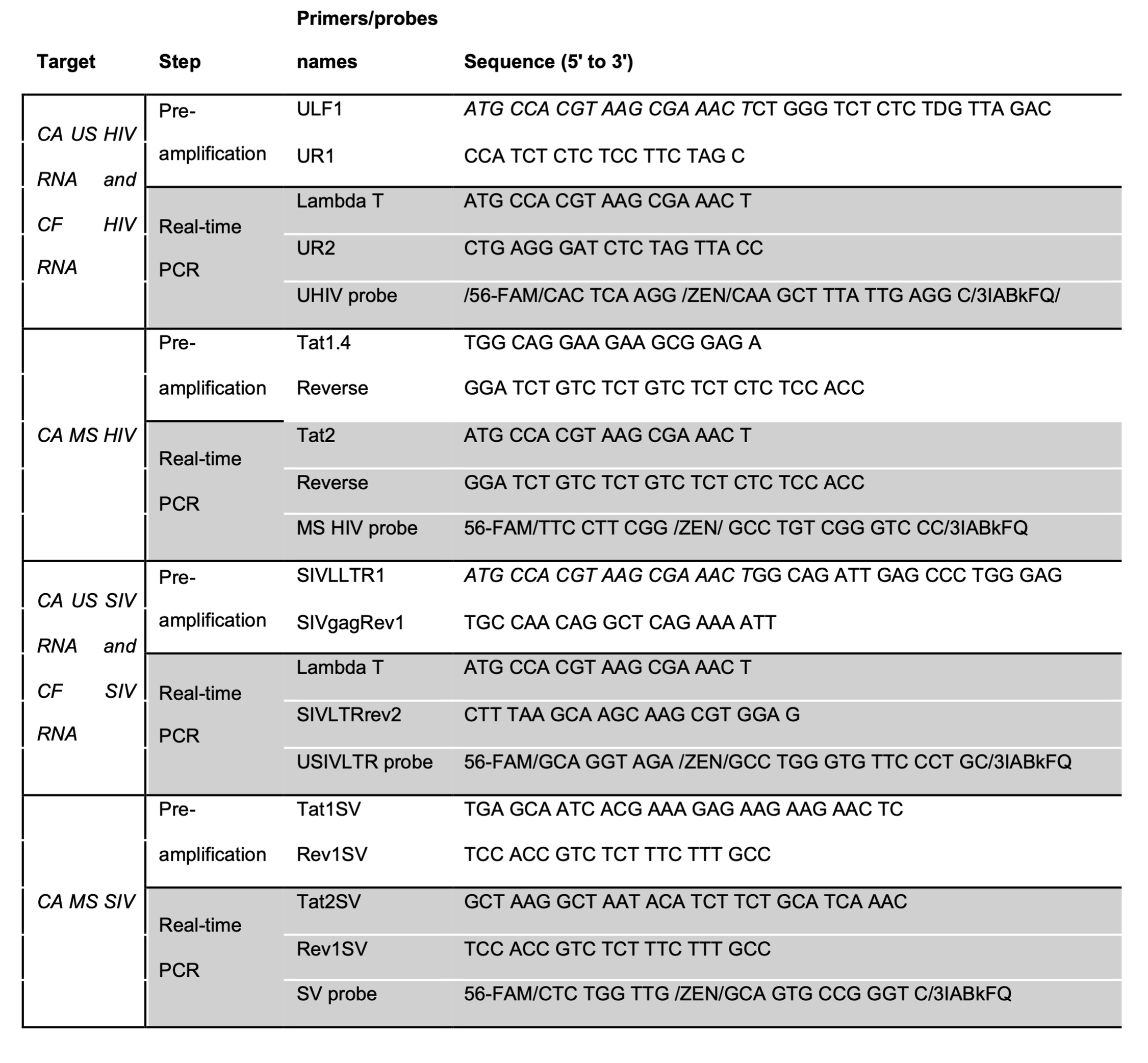

Supplement: S4 Table — (TIF) [file ppat.1010245.s004.tif]

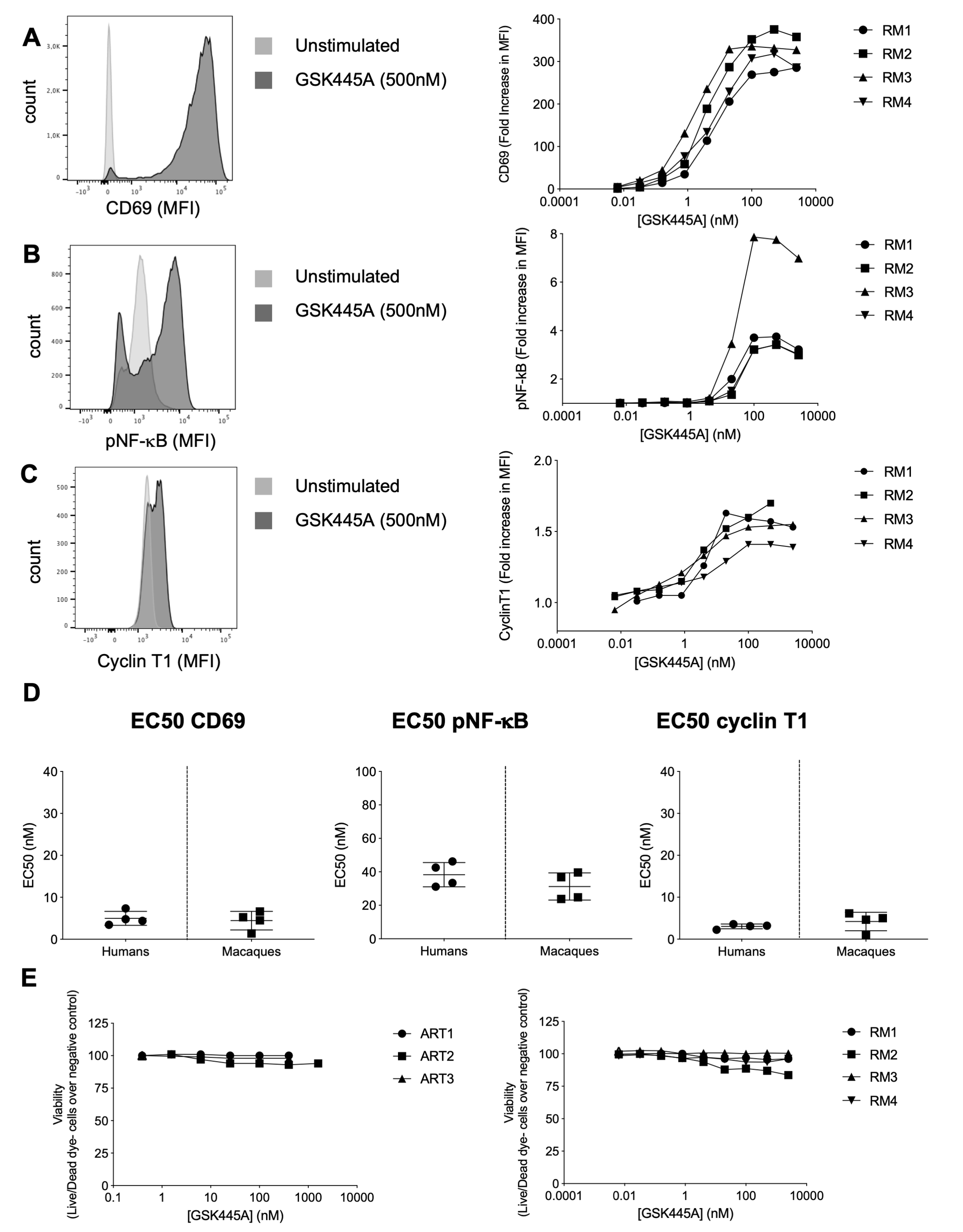

Supplement: S1 Fig — Expressions of cell surface CD69 (A), intracellular p-NFκB (B) and intracellular cyclin T1 (C) following stimulation with increasing doses of GSK445A were measured by flow cytometry in CD4+ T cells from 4 RM. Representative histograms (left panels) and dose response curves (right panels) are shown. D: EC50 of GSK5445A for induction of CD69, pNF-κB and cyclin T1. (E) Cellular toxicity of GSK445A was evaluated by exposing increasing doses of the compound to isolated CD4+ T cells from 3 virally suppressed individuals (left panel) and 4 RM (right panel). (TIF) [file ppat.1010245.s005.tif]

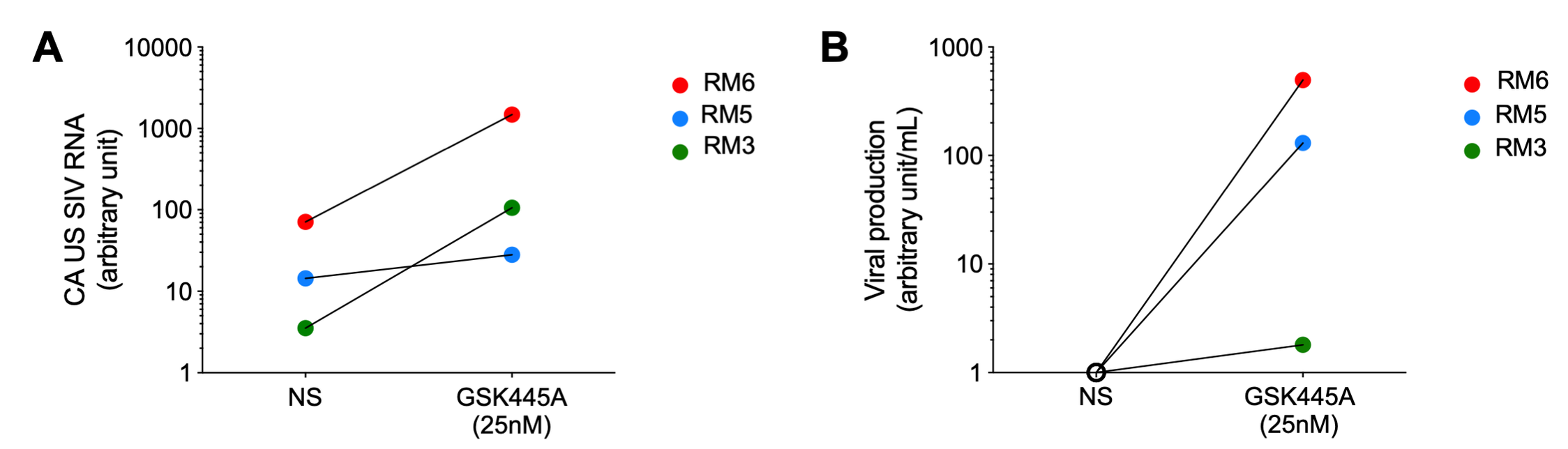

Supplement: S2 Fig — (A) Unspliced SIV RNA (gag) in CD4+ T cells obtained from 3 virally suppressed RMs and stimulated without (NS) or with GSK445A (25 nM). (B) Viral production in culture supernatants of CD4+ T cells obtained from 3 virally suppressed RMs and stimulated without (NS) or with GSK445A (25 nM). (TIF) [file ppat.1010245.s006.tif]

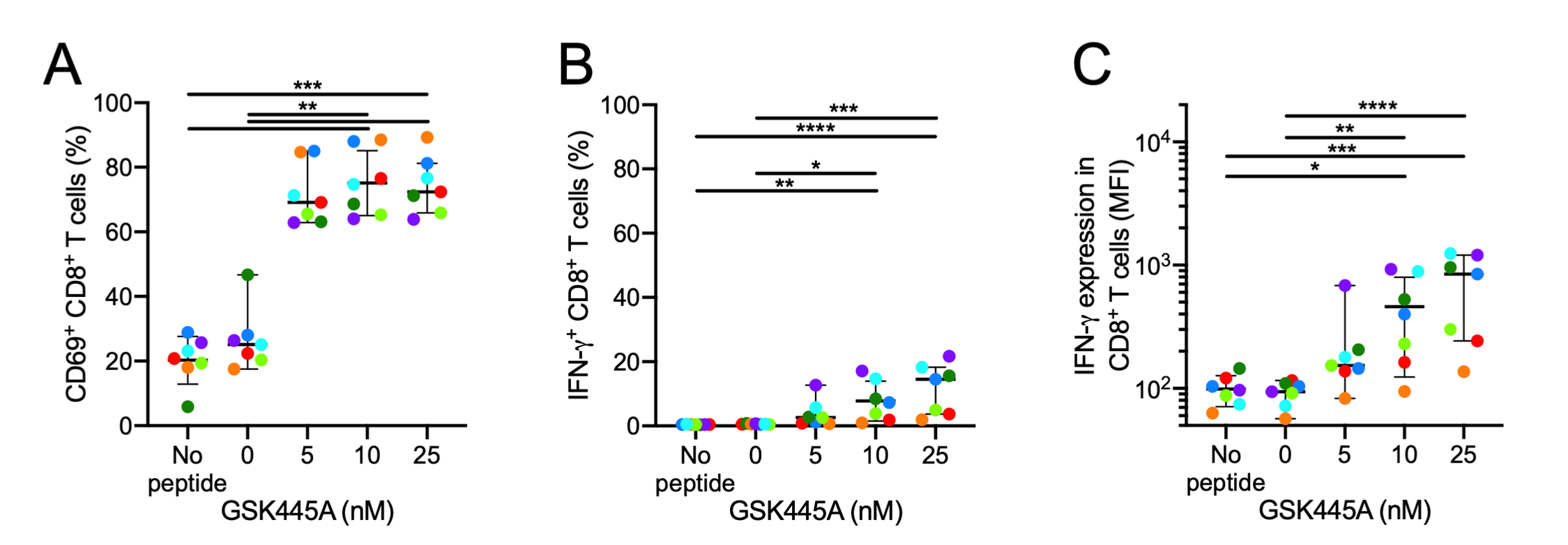

Supplement: S3 Fig — (A) CD69 expression on tetramer- total CD8+ T cells from 7 PWH after 6 hours of peptides stimulation with prior GSK445A pulse. Percentage of IFN-γ+ cells (B) and expression levels of IFN-γ (C) within total CD8+ T cells. Differences among conditions were analyzed by Friedman test. *P< 0.05; **P< 0.01; ***P< 0.001; ****P< 0.0001. (TIF) [file ppat.1010245.s007.tif]

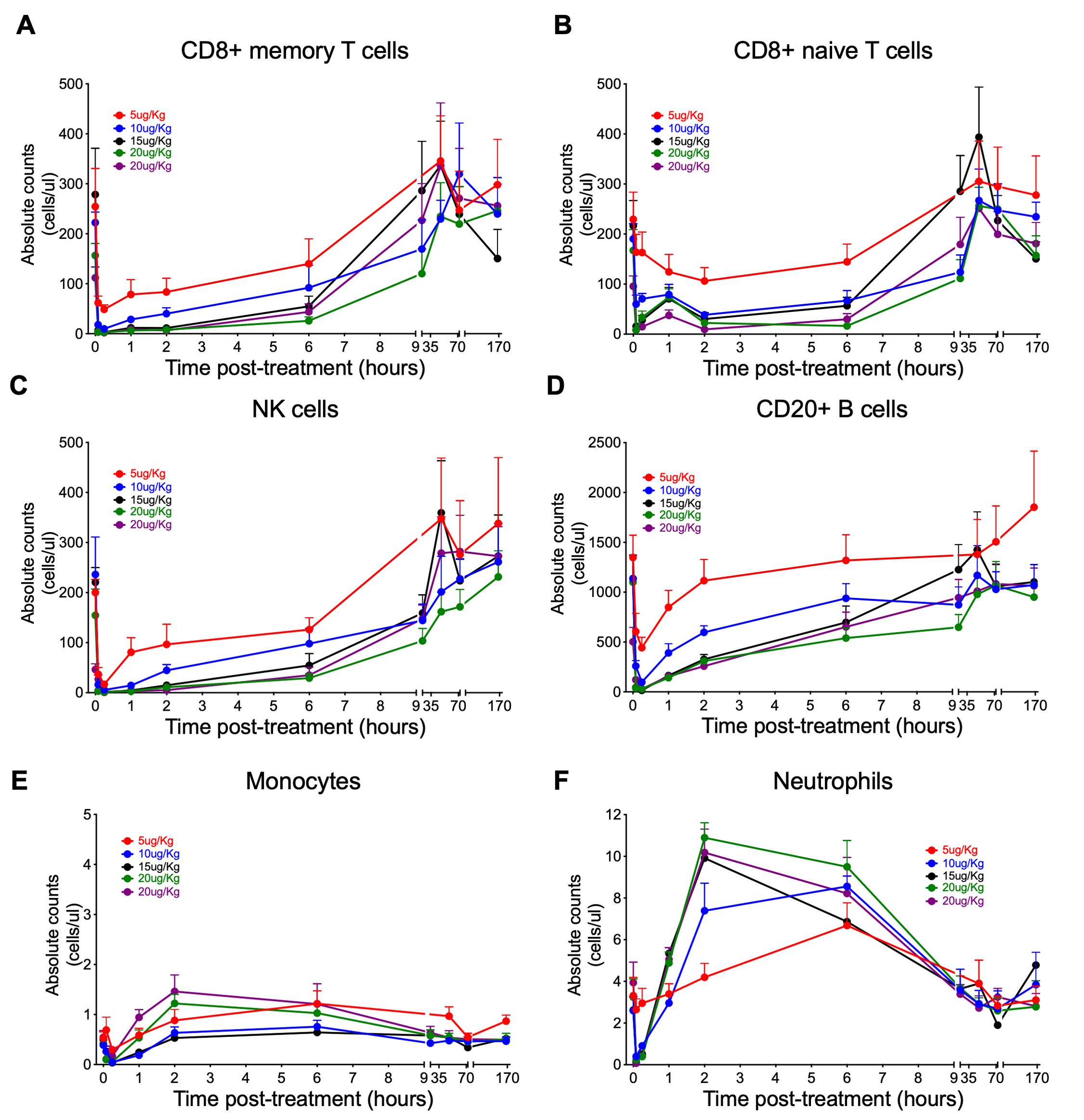

Supplement: S4 Fig — (A-F) Mean (+SEM) absolute counts of CD8+ memory T cells, CD8+ naïve T cells, NK cells, CD20+ B cells, monocytes and neutrophils in blood of RM (n = 5) following sequential intravenous infusions of GSK445A at 5 μg/kg, 10 μg/kg, 20 μg/kg, 20 μg/kg and 15 μg/kg at 14-day intervals. (TIF) [file ppat.1010245.s008.tif]

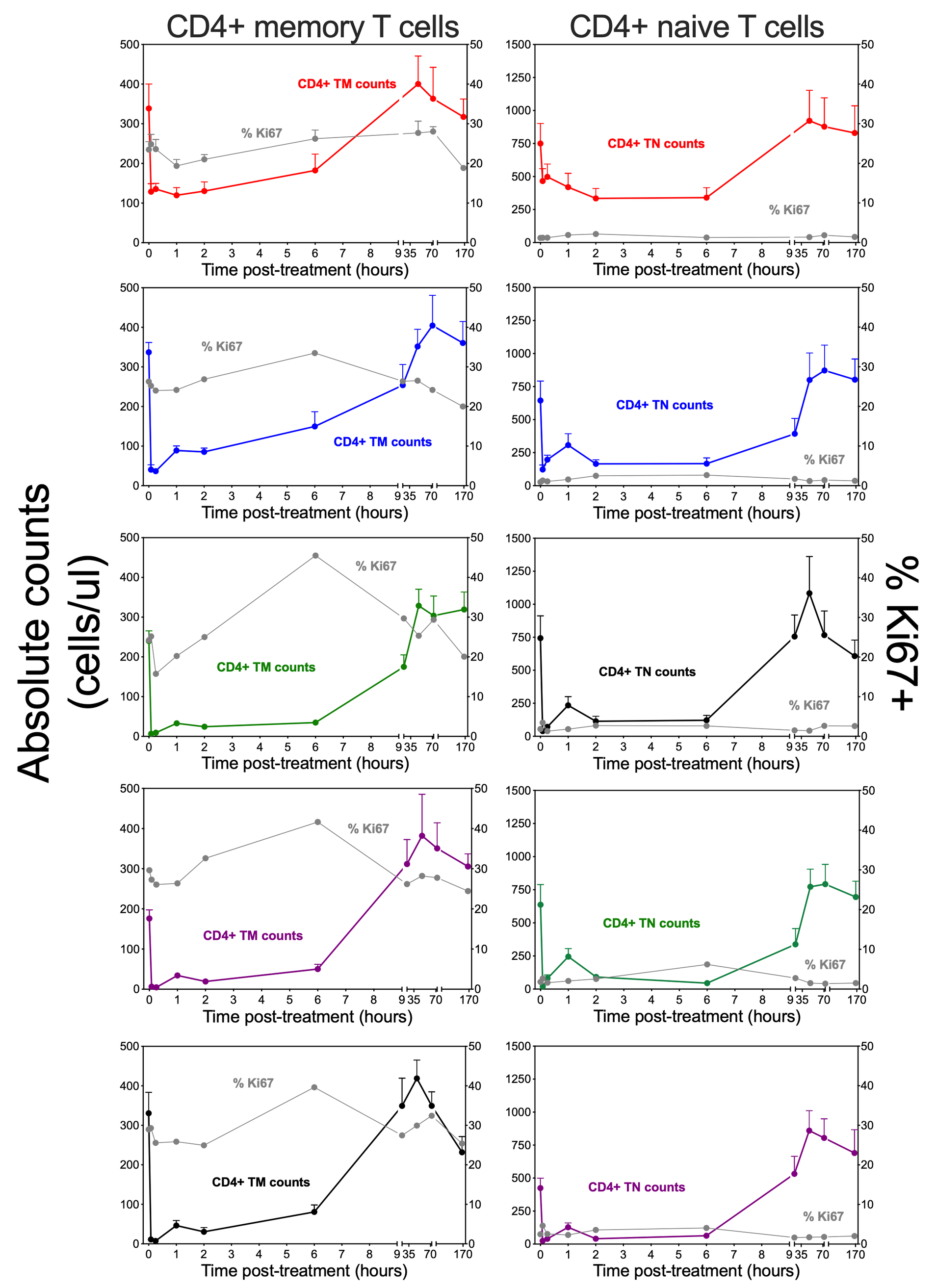

Supplement: S5 Fig — Absolute counts and frequencies of Ki67+ CD4+ memory T cells (left panels) and Ki67+ CD4+ naïve T cells (right panels) in blood of RM following sequential intravenous infusions of GSK445A at 5 μg/kg, 10 μg/kg, 20 μg/kg, 20 μg/kg and 15 μg/kg at 14-day intervals. (TIF) [file ppat.1010245.s009.tif]

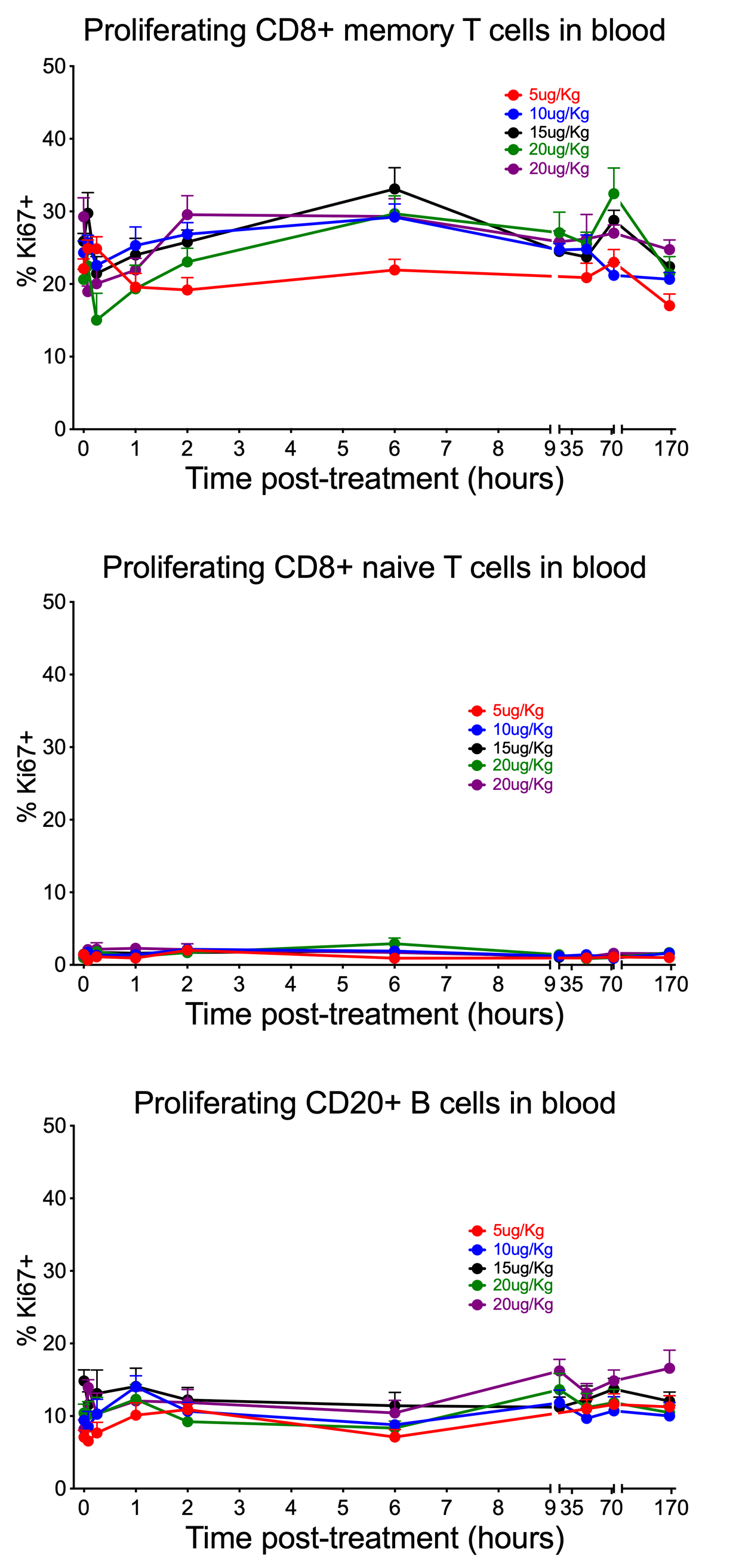

Supplement: S6 Fig — Mean (+SEM) frequencies of Ki67+ CD8+ memory T cells, Ki67+ CD8+ naïve T cells and Ki67+ CD20+ B cells in blood of RM (n = 5) following sequential intravenous infusions of GSK445A at 5 μg/kg, 10 μg/kg, 20 μg/kg, 20 μg/kg and 15 μg/kg at 14-day intervals. (TIF) [file ppat.1010245.s010.tif]

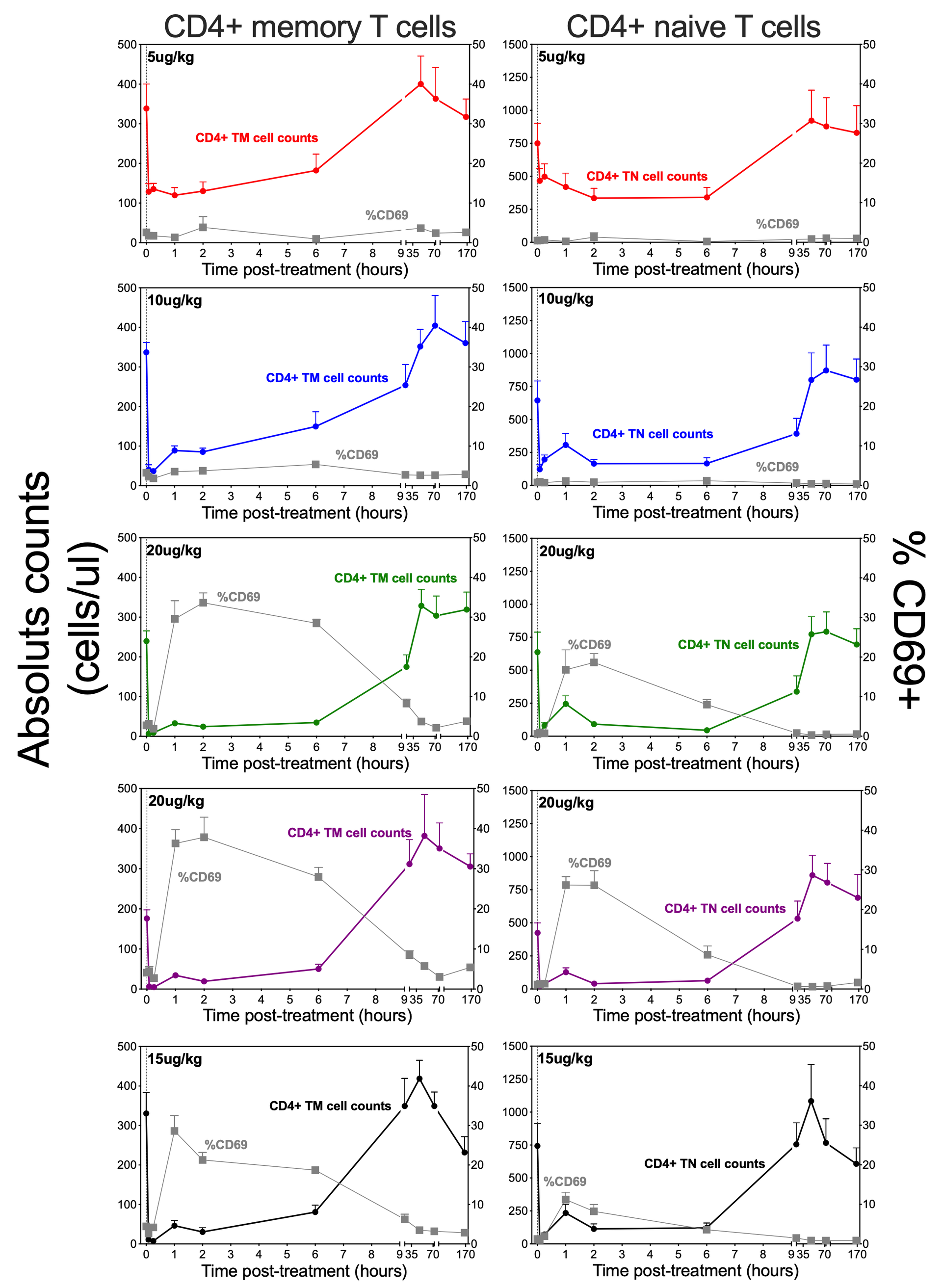

Supplement: S7 Fig — Absolute counts and frequencies of CD69+ CD4+ memory T cells (left panels) and CD69+ CD4+ naïve T cells (right panels) in blood of RM following sequential intravenous infusions of GSK445A at 5 μg/kg, 10 μg/kg, 20 μg/kg, 20 μg/kg and 15 μg/kg at 14-day intervals. (TIF) [file ppat.1010245.s011.tif]

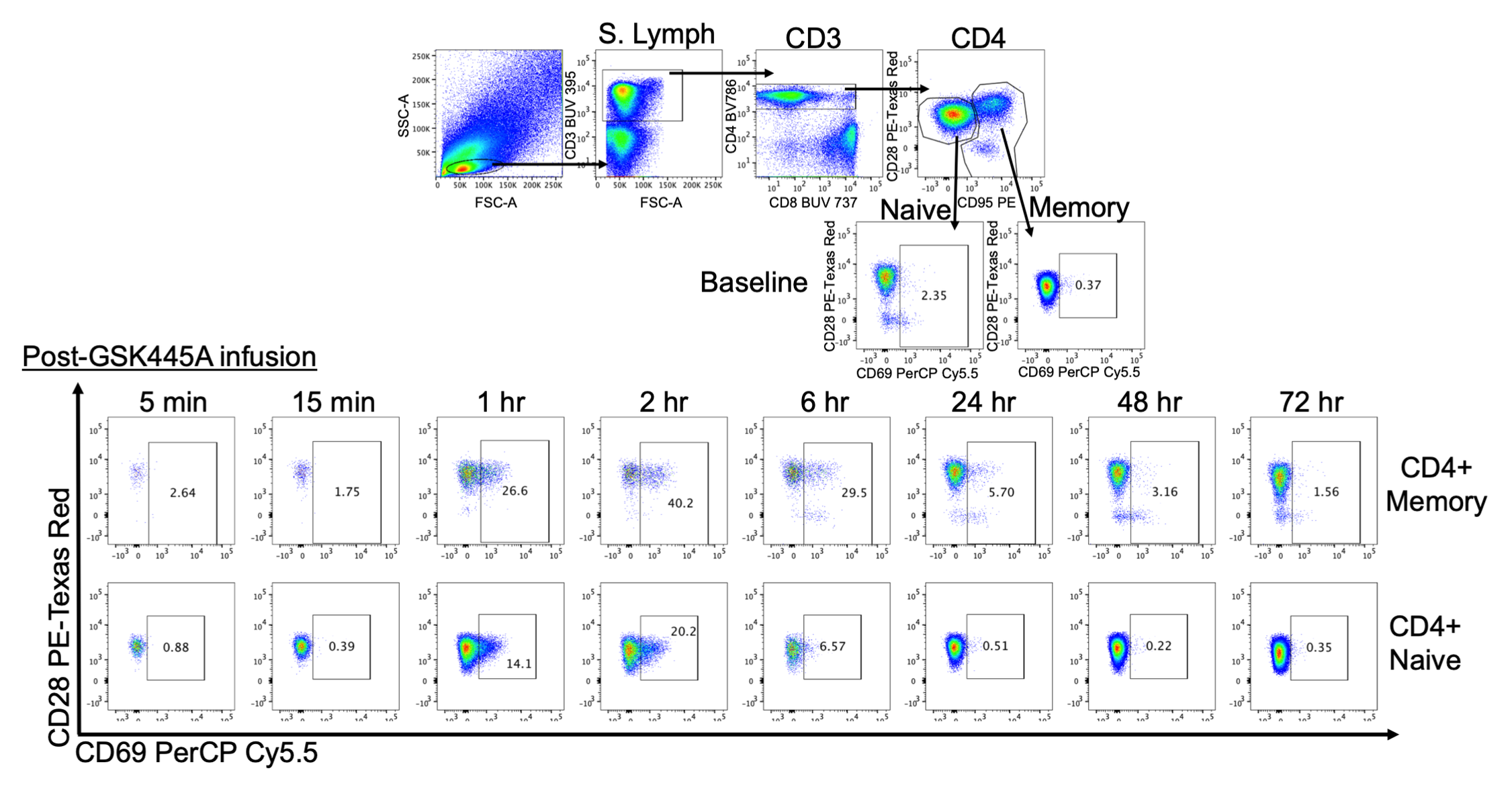

Supplement: S8 Fig — Representative flow cytometric analysis of an SIV-naïve RM showing CD69 expression on CD4+ memory and naïve T cells in peripheral blood following infusion of GSK445A at 20 μg/kg. (TIF) [file ppat.1010245.s012.tif]

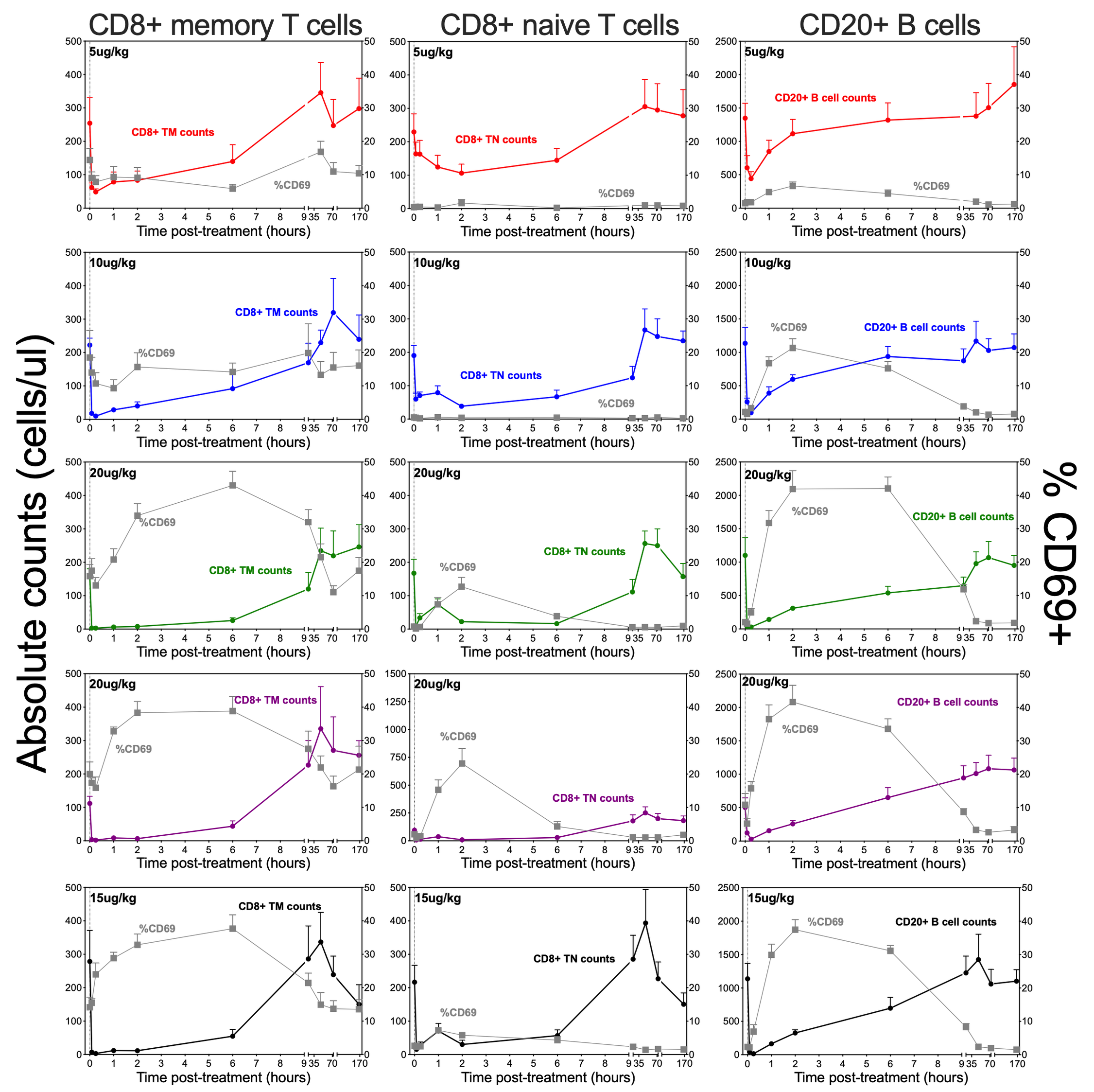

Supplement: S9 Fig — Absolute counts and frequencies of CD69+ CD8+ memory T cells (left panels), CD69+ CD8+ naïve T cells (middle panels) and CD69+ CD20+ B cells (right panels) in blood of RM following sequential intravenous infusions of GSK445A at 5 μg/kg, 10 μg/kg, 20 μg/kg, 20 μg/kg and 15 μg/kg at 14-day intervals. (TIF) [file ppat.1010245.s013.tif]

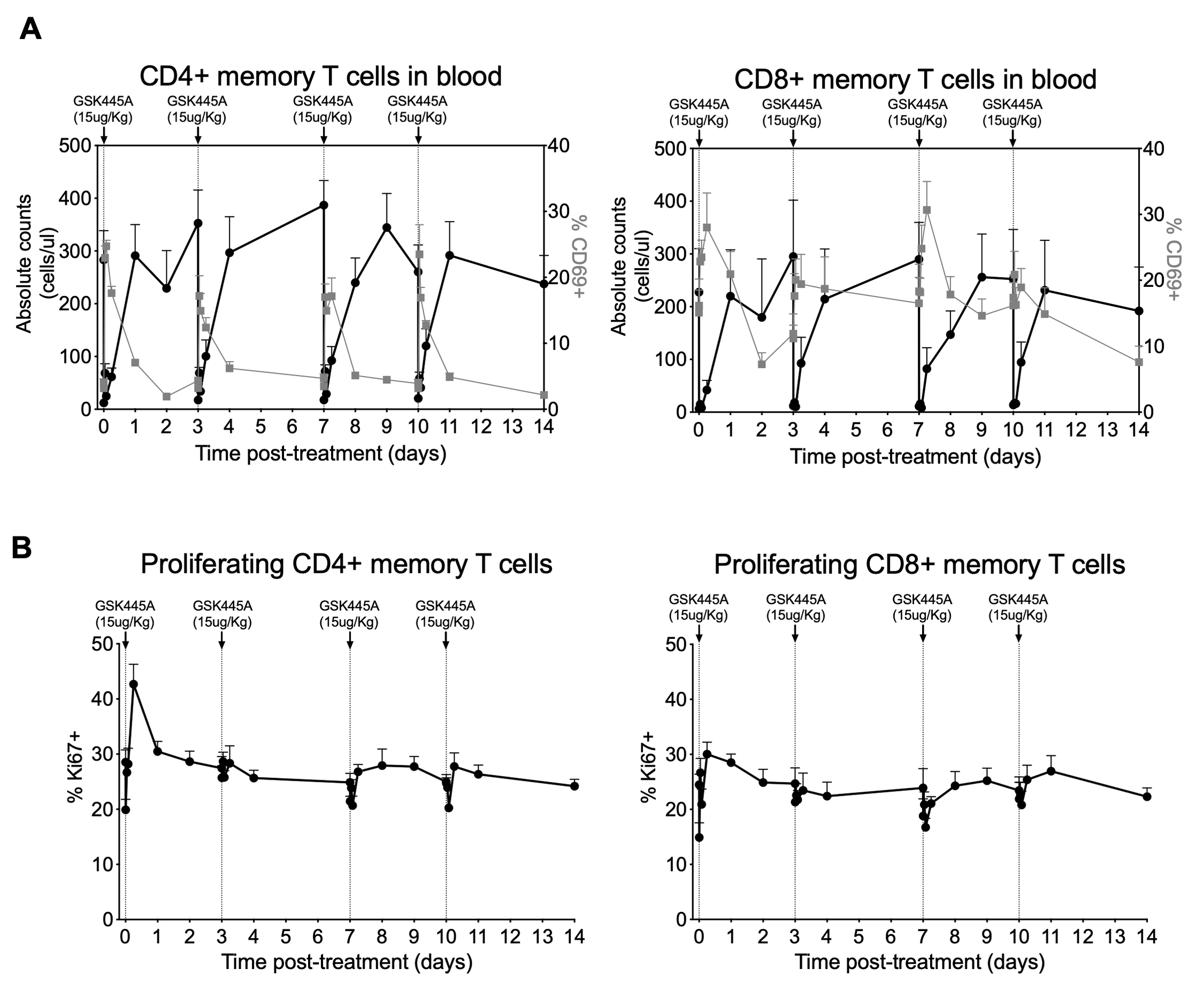

Supplement: S10 Fig — (A) Mean (+SEM) absolute counts and frequencies of CD69+ CD4+ memory T cells (left panel) and CD69+ CD8+ memory T cells (right panel) in blood of RM (n = 5) following 4 biweekly doses of GSK445A at 15 μg/kg. (B) Mean (+SEM) frequencies Ki67+ CD4+ memory T cells (left panel) and CD8+ memory T cells (right panel) in blood of RM (n = 5) following 4 biweekly doses of GSK445A at 15 μg/kg. (TIF) [file ppat.1010245.s014.tif]

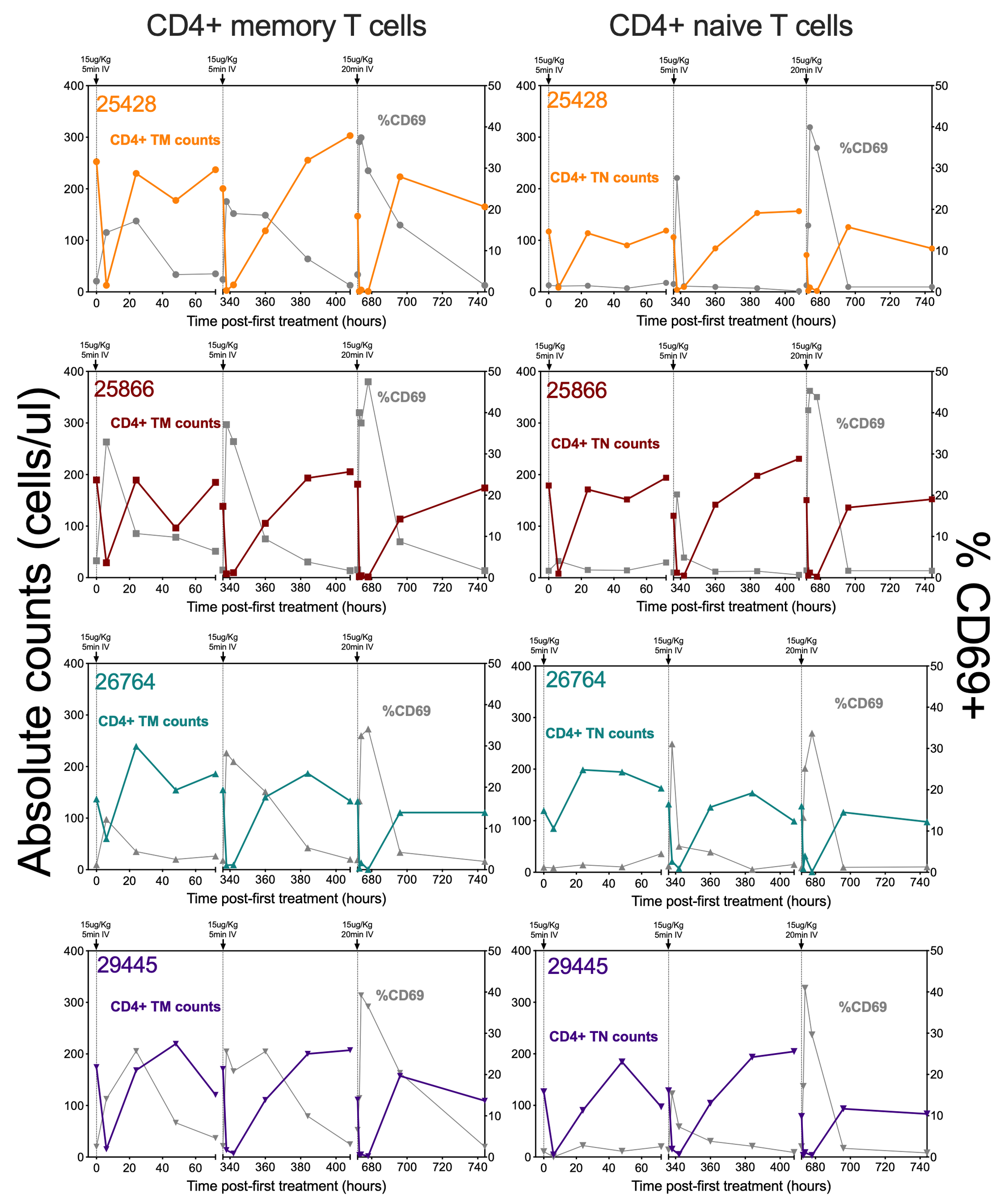

Supplement: S11 Fig — Absolute counts and frequencies of CD69+ CD4+ memory T cells (left panels) and CD69+ CD4+ naïve T cells (right panels) in blood of 4 SIV-infected RM on ART following 3 doses of GSK445A infusion at 15 μg/kg. (TIF) [file ppat.1010245.s015.tif]

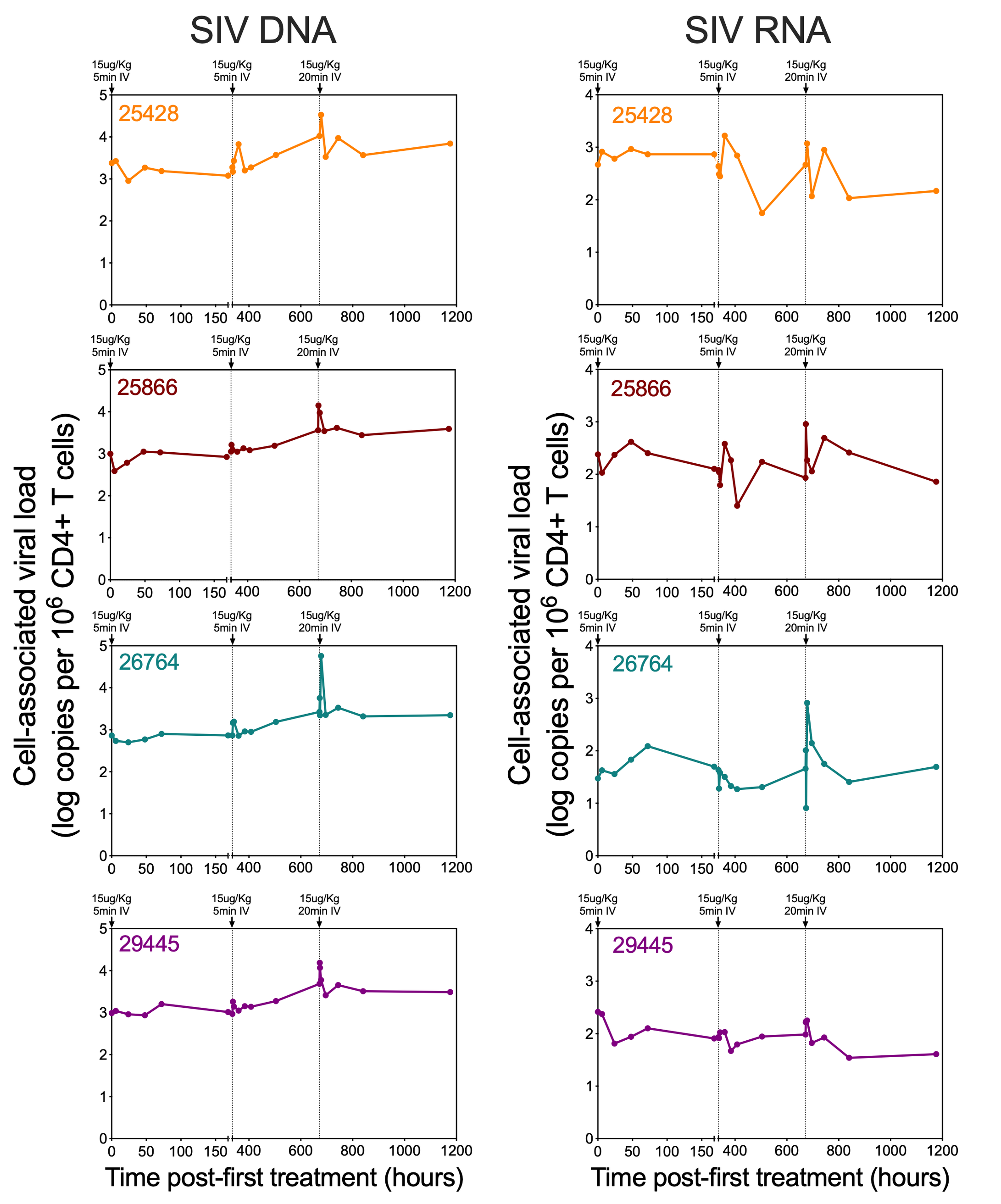

Supplement: S12 Fig — Cell-associated SIV DNA (left panel) and SIV RNA (right panel) in PBMC normalized to CD4+ T cells in each sample analyzed, from 4 SIV-infected RM on ART following 3 doses of GSK445A at 15 μg/kg. Cell-associated SIV RNA and SIV DNA were assessed by qRT-PCR and qPCR, respectively. (TIF) [file ppat.1010245.s016.tif]
